# The first checklist of alien vascular plants of Kyrgyzstan, with new records and critical evaluation of earlier data. Contribution 2

**DOI:** 10.3897/BDJ.10.e80804

**Published:** 2022-03-24

**Authors:** Alexander Sennikov, Georgy Lazkov

**Affiliations:** 1 Komarov Botanical Institute, Saint-Petersburg, Russia Komarov Botanical Institute Saint-Petersburg Russia; 2 University of Helsinki, Helsinki, Finland University of Helsinki Helsinki Finland; 3 Institute of Biology, Bishkek, Kyrgyzstan Institute of Biology Bishkek Kyrgyzstan

**Keywords:** Asteraceae, *Bidensfrondosa*, Central Asia, established aliens, *Hemerocallisfulva*, introduction, naturalisation, non-native plants, *Physalis*, plant invasions, Solanaceae

## Abstract

**Background:**

We continue the inventory of alien vascular plants of Kyrgyzstan, with emphasis on the time and pathways of introduction of the species and their current status in the territory. Each taxon is discussed in the context of plant invasions in Central Asia. This work is a further development of the preliminary checklist of alien plants of Kyrgyzstan, which was compiled for the Global Register of Introduced and Invasive Species in 2018.

**New information:**

This contribution includes all alien species of Kyrgyzstan belonging to Solanaceae and Asphodelaceae and one species of Asteraceae. *Physalisphiladelphicus* (syn. *P.ixocarpa*) is reported for the first time from Central Asia, as new to Kazakhstan, Kyrgyzstan and Uzbekistan, thus marking a recent invasion with a variety of imported grain and seed material. The old records of *P.ixocarpa* from Uzbekistan are based on misidentified specimens of *P.angulata*. *Physalisangulata* is an old cotton immigrant in Central Asia, whose invasion started in the 1920s; it is excluded from the alien flora of Kyrgyzstan as registered in error on the basis of cultivated plants. *Alkekengiofficinarum* is an archaeophyte of the Neolithic period in Central Asia, formerly used for food, now strongly declining and largely casual in Kyrgyzstan. The only historical record of *Physalisviscosa* from Uzbekistan was based on a technical error and belongs to *A.officinarum*. *Daturastramonium* and *Hyoscyamusniger* were introduced as medicinal plants during the period of the Arabic invasion of Central Asia, by the 11th century. *Daturainnoxia* is a newly recorded casual alien, recently escaped from ornamental cultivation. *Nicandraphysalodes* is a casual alien, which was cultivated by Russian colonists in the early 20^th^ century for culinary use and is currently used in ornamental cultivation. *Hemerocallisfulva* was a remnant of historical cultivation in the former Khanate of Buxoro, and its formerly established colonies are presumably extinct in the wild. *Bidensfrondosa* was seemingly introduced with contaminated forage and seed of American origin during the late Soviet period and started to spread in the period of independence; its invasion in the former USSR is analysed.

## Introduction

In their review of the data used in the analyses of alien plants, Hulme and Weser (2011) noted that the conclusions drawn from the databases of alien plants are highly dependent on the quality and completeness of the background data. So far, Central Asia in general and Kyrgyzstan in particular are nearly or totally omitted from the global database of naturalised alien plants (van Kleunen et al. 2018b). Aiming to overcome this striking deficiency, we have recently started developing a detailed checklist of non-native vascular plants of Kyrgyzstan, which includes the complete distributional data, the historical information on the time and pathways of introduction, and the actual status of invasion of a certain species (Sennikov and Lazkov 2021).

The current list of alien vascular plants of Kyrgyzstan (Sennikov et al. 2021) includes 184 species. Even though this list is deemed complete and accurate, it lacks the detailed distributional information and the analytical data, which remain unpublished for most of the species included. Our checklist is the place to collect, verify and evaluate those data, and to make them publicly available.

As in the first part of these contributions (Sennikov and Lazkov 2021), all occurrences in Central Asia are discussed in order to provide a solid background. This approach helps to uncover common pathways and periods of introduction and to trace and distinguish exceptional cases that do not fit the common patterns. The species discussions have a strong emphasis on the history of plant invasions in Central Asia and Kyrgyzstan, in order to link particular records with certain events in the political and social history.

The key plant family in the present contribution is Solanaceae, whose members have been completely inventoried for this purpose. This family is rich in alien plants; it concludes the top-10 of the families most represented in the global naturalised alien flora (Pyšek et al. 2017) and contains a number of critical species with either very old or quite recent history of introduction. The latest inventory of *Physalis* s.l. in Uzbekistan (Khassanov et al. 2020) demonstrated that its diversity is considerable but remarkably understudied. Our work aims to correct the misidentifications and uncover the timing of individual invasions and the processes that were driving the invasions of *Physalis* s.l. to Central Asia. Other genera were revised for completeness, i.e. *Datura*, *Hyoscyamus*, *Nicandra* and *Solanum*.

Besides the Solanaceae, we also included one rare alien, *Hemerocallisfulva* (Asphodelaceae), due to the extreme obscurity of its background data in Central Asian treatments. The recent expansion of a globally invasive weed, *Bidensfrondosa* (Asteraceae), has been largely neglected in Central Asia and is treated in detail here.

## Materials and methods

The checklist is alphabetically organised (according to genera and species) and structured according to Sennikov and Lazkov (2021). The emphasis is placed on the time and pathways of introduction and the current status and impact of certain species in Kyrgyzstan, in the context of plant invasions in Central Asia or the former USSR as a whole.

The study is largely based on herbarium specimens from Kyrgyzstan and Central Asia, which are kept at FRU, H, LE, MW and TASH. Personal herbarium collections of the authors have been deposited at H (A. Sennikov), FRU and LE (G. Lazkov). Besides, documented field observations published on citizen-science online resources (iNaturalist 2021, Plantarium 2021) have been used, together with documented and undocumented field observations made by the authors.

The set of all the records collected for the present work was included in the dataset of occurrences of alien vascular plants of Kyrgyzstan, which was published through GBIF (Sennikov and Lazkov 2022). Distributional maps were produced on the basis of these records.

**Figure 1.**
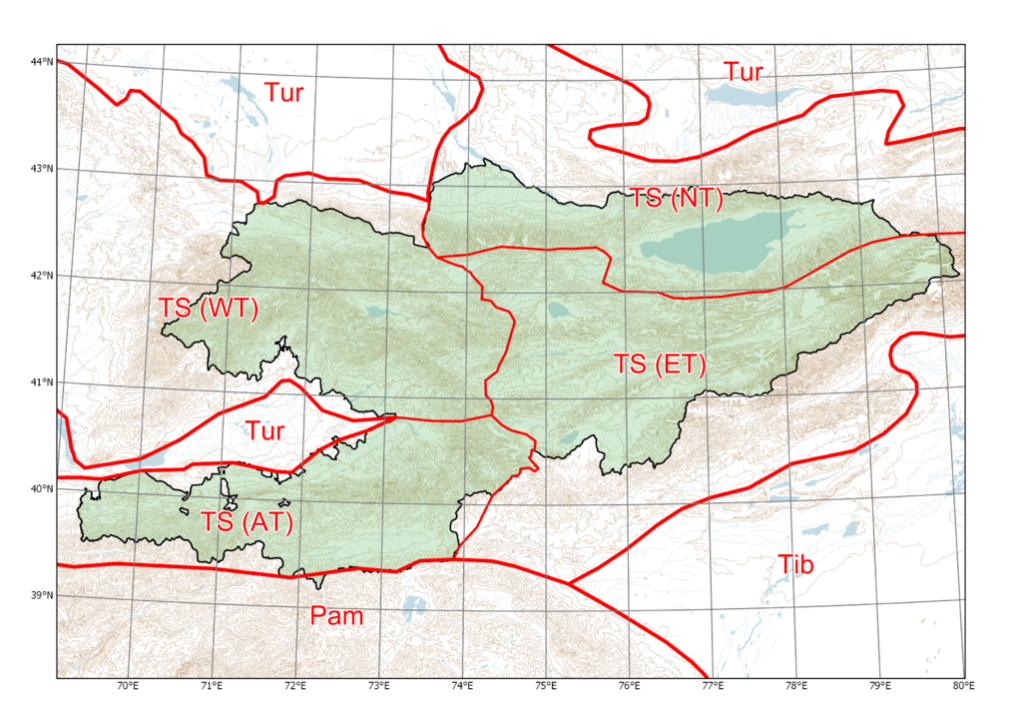
Major phytogeographic regions of Kyrgyzstan. Divisions (thick lines): TS (Tian-Shan), Tib (Tibet), Tur (Turanian), Pam (Pamir). Subdivisions (thin lines): AT (Alay-Turkestan), ET (Eastern Tian-Shan), NT (Northern Tian-Shan), WT (Western Tian-Shan). Source: Sennikov and Lazkov (2021).

Species distributions in Kyrgyzstan are characterised according to our scheme of botanical regions (Fig. 1). Species distributions outside Central Asia are given after PoWo (2021) and various taxonomic and floristic authorities.

The pathways of introduction are formalised according to Hulme et al. (2008) and Harrower et al. (2018). Concerning the invasive status, we accept the classification proposed by Richardson et al. (2000) and Pyšek et al. (2004). Species dynamics are observed or inferred from the past (50-100 years ago) and current (the latest 20 years) distributional data, and expressed as decreasing, stable or increasing without quantification.

## Taxon treatments

### Alkekengi officinarum

Moench, 1802

C7D89800-811F-5A91-857F-380BDD0F15BF

urn:lsid:ipni.org:names:814247-1

Alkekengi officinarum Moench, Suppl. Meth.: 177 (1802) — *Physalisalkekengi* L., Sp. Pl. 1: 183 (1753).
Alkekengi officinarum *Physalisfranchetii*Alkekengiofficinarumvar.franchetii
Alkekengi officinarum *Physalispraetermissa*
Alkekengi officinarum *Physalisglabripes*

#### Distribution

##### Native distribution

Many popular sources and even scientific data aggregators, including Plants of the World Online (PoWo 2021), stated that this species is native to Eurasia with the continuous distribution from Portugal to Japan. Palaeobotanical data definitely show that the species was present in Europe as early as in Pliocene (Särkinen et al. 2013), but this evidence does not indicate its continuous presence in the territory. As evident from the details of its distribution in particular countries, the species is native in two disjunct areas: the Caucasus (Grossheim 1967) and central China (Li 1973) with adjacent territories.

##### Secondary distribution

The species was a common vegetable in pre-historic times (Colledge and Conolly 2014). For this reason, it had been transported with people as they settled in new territories since the Neolithic period (e.g. Kohler-Schneider and Caneppele 2007, Reed 2015, Jin et al. 2020). With humans, it expanded as an archaeophyte to Europe, Central Asia and neighbouring mountainous areas (including Xinjiang). Its occurrence in the Russian Far East (Ignatov 1991) originated from the ancient Chinese colonisation (Schischkin 1936). Its non-native status in Central Asia was established by Pojarkova (1954a).

The species is a neophyte outside Eurasia, in North America and northern Africa.

##### Distribution in Central Asia

The species is widely distributed in Central Asia and has been recorded from every country of the region (Kovalevskaya 1987). It was commonly cultivated before the Russian colonisation (Fedtschenko and Fedtschenko 1913) and occurred spontaneously in gardens and around populated places.

Due to a technical error, *P.viscosa* L. was reported as historically occurring in Uzbekistan (Khassanov et al. 2020). This record was based on a misfiled collection of *A.officinarum* from Tashkent (cultivated or weedy), dated 1919.

##### Distribution in Kyrgyzstan

Western Tian-Shan, Northern Tian-Shan, Alay-Turkestan.

**Figure 2.**
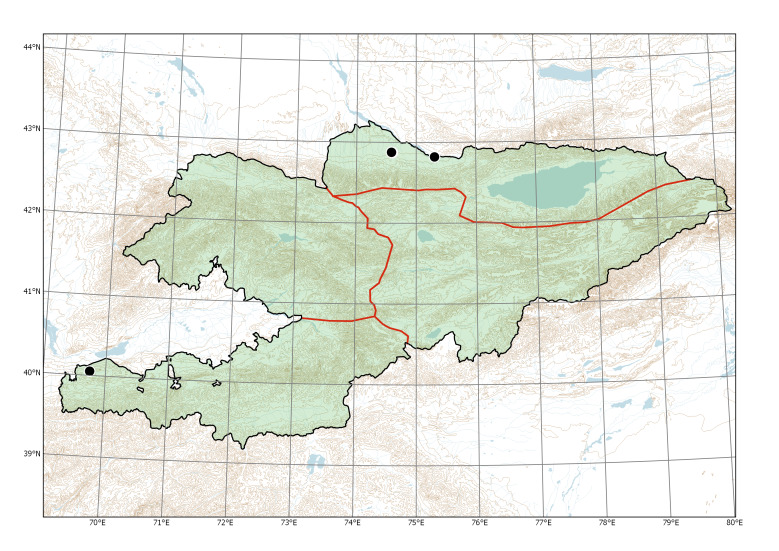
Recorded distribution of *Alkekengiofficinarum* in Kyrgyzstan, according to historical specimens examined (cultivated plants excluded) and recent observations.

The species has been commonly observed in and around populated places, along irrigation ditches and field margins. It was commonly cultivated in the whole country (Spota 1960) but went out of fashion and became rare nowadays (Lazkov, pers. obs.). Historical specimens do not provide any reliable data on its former distribution (Fig. 2); we assume that the cultivation was concentrated in climatically favourable, agricultural areas of western and northern Kyrgyzstan.

#### Ecology

Riversides in moist forests in the native distribution area; cultivated lands, sides of watercourses, humid forests in the secondary distribution area.

#### Biology

Perennial, rhizomatous, spreading by rhizome growth, persisting for a long time without seed reproduction.

#### Notes

The disjunct native distribution of the species in Eurasia is reflected in its infraspecific variability and, consequently, in its synonymy. Pojarkova (1954a) recognised that plants from the eastern (Chinese) part of the distribution area largely differ in subglabrous leaves, calyces and pedicels, and established a few species-level segregates to reflect this observation. She mostly referred Central Asian plants to *P.praetermissa*, a subglabrous variant of *P.alkekengi* with its centre of distribution in China, thus indicating their human-dispersed origin from that country. Latest taxonomic treatments (e.g. Zhang et al. 1994) did not support this splitting, leaving the species as the sole member of the genus *Alkekengi*, a generic segregate related to *Physalis* (e.g. Whitson and Manos 2005, Zamora-Tavares et al. 2016). Since both subglabrous and hairy variants of *A.officinarum* are present extensively in China and Central Asia (Vasilieva 1965, Zhang et al. 1994), these variants are currently treated at the level of variety, as A.officinarumvar.franchetii (Zhang et al. 1994, Wang 2014).

#### Introduction to Kyrgyzstan

##### Period of introduction

Archaeophyte.

The species is an archaeophyte of the Neolithic period, which was introduced from China in pre-historic times. It has been grown in China for at least six thousand years (Jin et al. 2020) for its edible fruits (Li 1973) and is still consumed in some rural territories (e.g. Kang et al. 2013, Wang et al. 2020).

##### Pathways of introduction

Escape from confinement: Agriculture.

The species was introduced and originally used as a vegetable. When its role as a vegetable had decreased and was largely forgotten, it was still cultivated as an ornamental and traditional plant.

The species colonised the territory around the places of original cultivation by vegetative growth and seemingly by seed dispersal along water streams (cf. Cappers 1993). Whereas the species was frequently noted in walnut forests in the proximity of villages in Uzbekistan (Kovalevskaya 1961), no such wild occurrence is known in Kyrgyzstan, thus indicating that its seed dispersal was very limited or inefficient. Most likely, the main agent of its local dispersal was humans.

##### Source of introduction

China.

##### Invasion status

Largely casual (persisting in places of original cultivation) or locally established. All recent observations are from the places of former cultivation (Lazkov, pers. obs.), which should be treated as casual. Not invasive.

##### Evidence of impact

Agriculture - no impact (the species currently does not occur as a weed, although it was formerly recorded along fields: Spota 1960). Native ecosystems - no impact (not occurring in native habitats). Urban areas - minor impact (may occur as a ruderal in populated places when the cultivation was abandoned).

##### Trend

**Figure 3.**
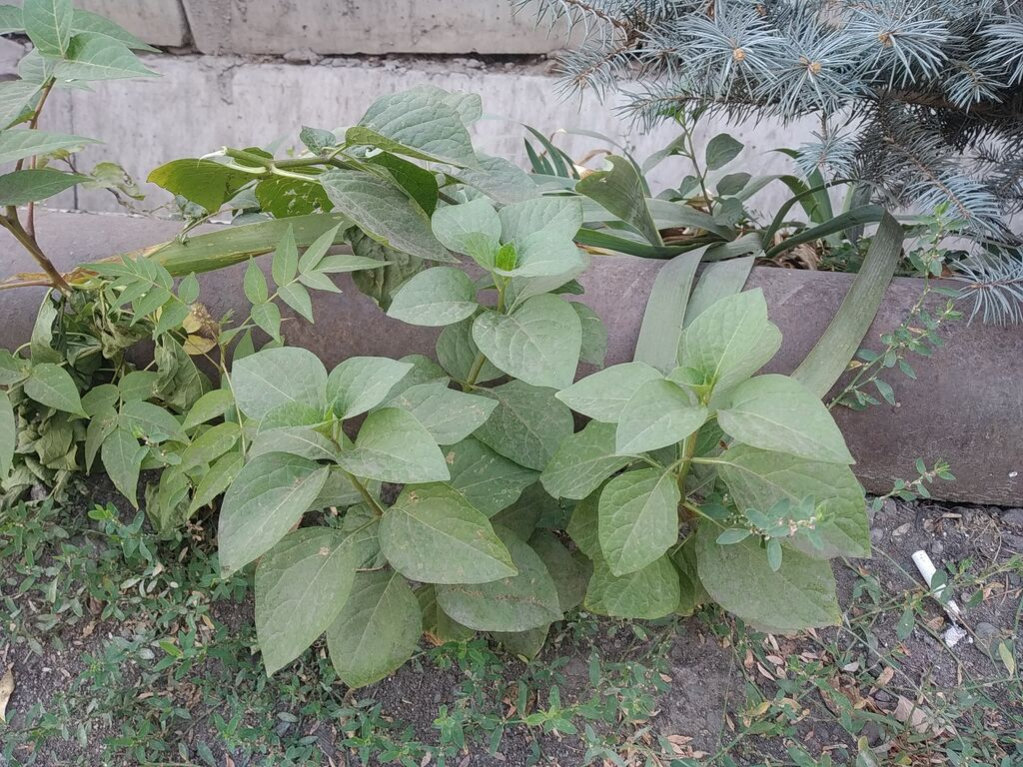
*Alkekengiofficinarum*, a survivor of long-abandoned cultivation in Bishkek (photo by G. Lazkov, 21 September 2021).

Strongly decreasing. The species had been very common in agricultural areas and, at that time, was commonly observed around populated places (Spota 1960). When the tradition of the species cultivation had practically ceased, it disappeared or much decreased in many places and can be rarely seen nowadays (Fig. 3); this observation evidences that the species largely relied on cultivation for its persistence.

### Bidens frondosa

L. 1753

396193E6-A589-579A-991D-FB87E990FA88

urn:lsid:ipni.org:names:315743-2

Bidens frondosa L., Sp. Pl. 2: 832 (1753).
Bidens frondosa *Bidensmelanocarpa*

#### Diagnosis

The species differs from *Bidenstripartita* L., which is common in Central Asia (Nabiev 1993) and Kyrgyzstan (Gorbunova 1965), in narrow, long-attenuated and narrowly petiolate leaflets, and in two (vs. 3-4) setae on the achenes. In the beginning of its invasion, it has been commonly confused with the latter species, thus obscuring the data on its actual occurrence.

#### Distribution

##### Native distribution

North America.

##### Secondary distribution

Europe, Asia (southern Siberia, Central Asia, Eastern Asia), Australia and New Zealand; sporadically also elsewhere.

In Europe, this species belongs to the most widely distributed alien vascular plants (Lambdon et al. 2008). It also belongs to the most invasive plants in Russia (Morozova and Vinogradova 2018) and Belarus (Dzhus 2020).

##### Distribution in Central Asia

Kazakhstan, Kyrgyzstan, Tajikistan, Uzbekistan.

In Central Asia, the species was first recorded in a single locality on the south-eastern margin of Tashkent City, Uzbekistan, in 1990 (Alexeev 1991). The species was found naturalised on irrigated grassland in newly developed city districts. By the beginning of the 2010s, the species was found commonly naturalised and invasive in agricultural areas of Uzbekistan (Maltsev 2013).

In Kazakhstan, the species was first recorded in 2001 near Jänıbek in West Kazakhstan Region. This locality is situated immediately next to the Russian border, and the species was known from the southern Volga Region of Russia by that time (Sukhorukov and Berezutsky 2000). Its current distribution seems to be quite wide, especially in agricultural areas of the north (Plantarium 2021).

The species was first recorded in Kyrgyzstan by Lazkov et al. (2011), based on a single observation in Bishkek dated 2008. More data are reported in the present Contribution.

In Tajikistan, the species was first recorded from Dushanbe City and its vicinities in 2009, along roadside ditches (Nobis and Nowak 2011). It was also found on rice fields near Hissar (Nowak et al. 2013).

At present the species is naturalised in all these four countries. It is widely naturalised and invasive in Kazakhstan and Uzbekistan, but sparsely occurring and not yet invasive in Kyrgyzstan and Tajikistan.

##### Distribution in Kyrgyzstan

**Figure 4.**
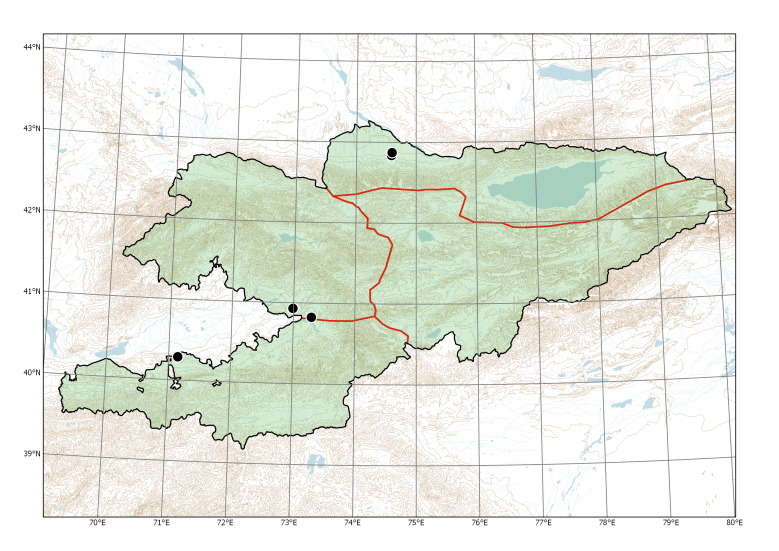
Currently recorded distribution of *Bidensfrondosa* in Kyrgyzstan.

Western Tian-Shan, Northern Tian-Shan, Alay-Turkestan (Fig. 4).

**Figure 5.**
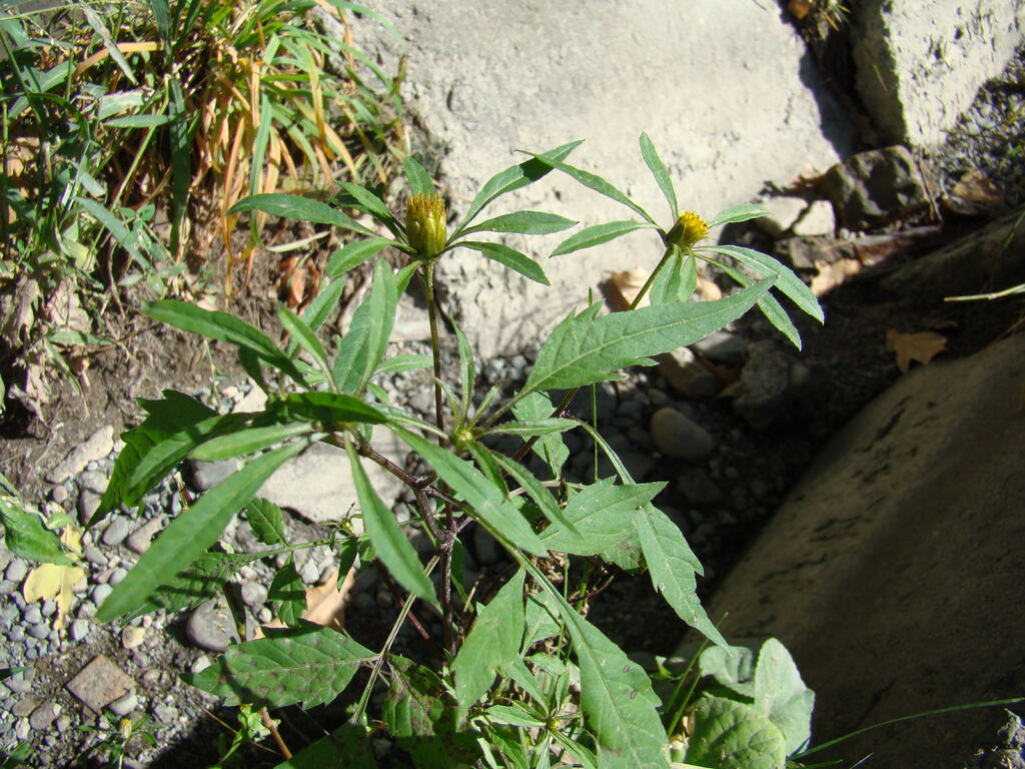
*Bidensfrondosa* in Bishkek, Kyrgyzstan (photo by G. Lazkov, 19 June 2018).

*Bidensfrondosa* was recorded in Bishkek in 2008, for the first time in Kyrgyzstan. Since then, small groups of the species have been observed in the city centre (Fig. 5). These occurrences are mostly ephemerous, not lasting long, but their regular re-appearance suggests that the invasion is continuous. The species was also observed established in the Botanical Garden, where it has found a suitable agricultural habitat and experiences little pressure from the environment.

During 2011-2020, we also observed small groups of *B.frondosa* in a few localities in the Fergana Depression, along the border with Uzbekistan. These previously unpublished records suggest that the species may be found elsewhere in the Depression because of its common naturalisation in Uzbekistan (Maltsev 2013).

So far, all the localities are from altitudes between 650 and 1000 m above sea level, and the species shows no tendency to spread to the mountains.

#### Ecology

Sides of water bodies and flood plains in the native distribution area; river sides, wetlands, fields and ruderal places in the secondary distribution area.

#### Biology

Annual.

*Bidensfrondosa* can grow taller than *B.tripartita*, producing more seeds, and, therefore, can outcompete the latter in agricultural and even native environments (Danuso et al. 2012). This process has been observed in many countries, for example, Uzbekistan (Maltsev 2013) and Russia (Glazkova 2005).

#### Taxon discussion

The species is highly variable in certain characters. Bidensfrondosavar.anomala was distinguished by the achenes antrorsely barbate along the whole margin, whereas achenes of the type variety are antrorsely barbate along the body but retrorsely barbate along the awns (Sherff 1937, Verloove 2021). Both varieties were recorded in Uzbekistan (Maltsev 2013). Variants with shorter and longer outer phyllaries were also observed in herbarium collections. These observations indicate a high genetic diversity and multiple events of the species' introduction to Central Asia, contrary to the hypothesis of Vinogradova et al. (2009) about a single founder effect in the East European invasion.

#### Notes

Rice has been commercially grown in the USA already in the 19th century (Barrett and Seaman 1980). In the early 20th century, *Bidensfrondosa* was a common weed of rice fields in California (Kennedy 1923), although later works do not list this genus at all (Barrett and Seaman 1980). The preference of *B.frondosa* for damp places accounts for its adaptability to rice fields; when introduced to Eastern Asia, the species became a noxious weed of rice fields in Korea (Oh et al. 2007) and north-eastern China (Zhu et al. 2020). It is also capable of infesting other crops, like maize, soybean and sugarbeet, in Italy (Danuso et al. 2012) but may be lacking on fields in other countries like in Germany (de Mol et al. 2015).

The attachment of *Bidensfrondosa* seed to agricultural commodities is indicated by its numerous records at mills and railway stations (Suominen 1979, Gudžinskas 1989; references in Glazkova 2005). Introduction with North American grain (maize, wheat or oat) was suggested in Finland (Suominen 1979). Besides railways, sea ports may act as entrance points for the species (Vorobiev 1954 and references in Glazkova 2005).

In Eastern Europe, the species was commonly recorded in many regions during the 1980s and 1990s (review in Glazkova 2005). However, the persons who recorded the species often noted its possible presence in the territory already for some considerable period, thus indicating that there was a significant backlog due to the superficial similarity of *B.frondosa* to the common East European native species *B.tripartita*. First records indicate the appearance of *B.frondosa* in towns and at railway stations as early as in 1955-1970 (Glazkova 2005). As the early species' records show a clear relationship with the transportation of grain and accompanying commodities, we assume that its original appearance in Eastern Europe was connected with the transportation of imported agricultural goods.

This import may have a long and complex history. The first record of *B.frondosa* on the railway in Brest was dated 1955 and can be linked with the transportation of grains from Poland, which was noticeable since 1953 (Mackie 1968). Further on, the species was recorded in Kirov Town, far away from possible sources in Europe, along a small river streaming through an industrial area with many railways (Glazkova 2005). This record may be connected with the import of American grain that followed the drought of 1963 (Zelenin 2014).

Due to the lack of early records in agricultural communities (on fields and field margins), we conclude that contamination of seed material was not a major pathway of the species' introduction into Eastern Europe, and it was contamination of imported forage (animal feed) and, to some extent, food (grain) that was responsible for the mass invasion of *B.frondosa* in the USSR.

In 1965, the USSR gave up the notorious corn campaign and started to import feed grain (first of all, maize) from the USA; further on, a vast amount of American feed grain had been imported since 1973 as a response to the decision to increase national food consumption and to maintain extensive livestock (Mackie 1968, Novotny and Shull 1985, Allen 1987). This event coincides with the rapid rise of *B.frondosa* in the European part of the USSR (Glazkova 2005), indicating that feed grain was the most likely source of the species' invasion. The coinciding increase in the abundance of *B.frondosa* in the 1970s-1980s was recorded in Belarus (Dzhus 2020). Corn was dominating in the global production and export of feed grain (Novotny and Shull 1985), and seeds of *B.frondosa* may be found harvested and transported together with that crop (James et al. 2015).

Besides Eastern Europe, the second major area of the species' invasion in Russia is the Far East (Morozova and Vinogradova 2018). The species arrived in that territory very early, being naturalised already by the beginning of the 1950s (Vorobiev 1954). Its invasion has likely started from the port areas, where the species was widespread in the 1990s (Barkalov 1992). Its active and continuous import with grain is therefore assumed.

The primary further dispersal of *Bidensfrondosa* seed in urban habitats may occur with the aid of Fringillidae birds, domesticated animals or humans.

The common occurrence of *Bidensfrondosa* along water streams suggests its further dispersal with water flows and transport. This type of dispersal was inferred for the first species' expansion in Central Europe (Hejný and Lhotská 1964, Lhotská 1966).

The fruits of *Bidensfrondosa* have two barbate awns, and their lateral margins are also barbate. This accounts for their ability to attach to the animal fur and feather, which, in the case of water birds, allows for successful dispersal of the species along aquatic habitats (Carlquist 1966). This pathway was seemingly a major factor in the recent species' expansion in Eastern Europe (Gudžinskas 1989, Glazkova 2005). The first occurrence of this kind was found at the estuary of the Dnestr River (Ukraine) already in 1968 (Glazkova 2005).

Besides exozoochory, another proven way of the species' dispersal by aquatic birds is endozoochorous; various duck species are known to eat its seed, thus aiding their further dispersal (Green et al. 2016). Water birds are apparently responsible for bringing the species to new localities, which are often hidden from even minor pathways of dispersal of weeds and ruderal plants (e.g. Leostrin et al. 2018).

#### Introduction to Kyrgyzstan

##### Period of introduction

Neophyte.

The first record of the species in Kyrgyzstan, dated 2008 (Lazkov et al. 2011), most likely does not reflect its first arrival in the territory. Taking into account its first observation in Uzbekistan, dated 1990 (Alexeev 1991), with its extensive naturalisation subsequently revealed in the beginning of the 2010s (Maltsev 2013), the introduction should have started from the late Soviet period, during the 1980s, if not earlier.

##### Pathways of introduction

Transport - Contaminant: Contaminated bait. Transport - Contaminant: Seed contaminant. Transport - Contaminant: Contaminant on animals.

The most likely pathway of introduction of *Bidensfrondosa* in Kyrgyzstan was its arrival with contaminated forage, but we also cannot exclude its appearance on corn fields as a seed contaminant. The occurrences along irrigation ditches may be zoochorous.

Further dispersal may occur with water, humans, domestic animals and water birds.

##### Invasion status

Naturalised.

Although most of the occurrences observed so far have been represented by just a few plants, and some were proven to have disappeared, the species is apparently on the way to its naturalisation in the country. It can be considered naturalised at least in the Botanical Garden in Bishkek.

No large populations or founder localities have been noticed so far.

##### Evidence of impact

Agriculture - minor impact (rarely occurring along irrigation ditches, once recorded as a garden weed). Native ecosystems - no impact (restricted to agricultural and urbanised areas). Urban areas - minor impact (rarely occurs in ruderal places).

##### Trend

Increasing (observed).

### Datura innoxia

Mill. 1768

A99C5A17-0E52-50A0-B6CB-76B3AC586405

urn:lsid:ipni.org:names:316945-2

Datura innoxia Mill., Gard. Dict., ed. 8: *Datura* no. 5 (1768).
Datura innoxia *Daturameteloides*
Datura innoxia *inoxia*Barkworth and Rabei 2020

#### Distribution

##### Native distribution

Central America.

##### Secondary distribution

North and South America, Europe, Africa, Southern Asia, Australia.

##### Distribution in Central Asia

Kyrgyzstan, Uzbekistan.

The species was recorded as a rare alien in Uzbekistan, observed as ruderal or escaped from cultivation (Kovalevskaya 1961).

Reported from Kyrgyzstan for the first time here.

##### Distribution in Kyrgyzstan

**Figure 6.**
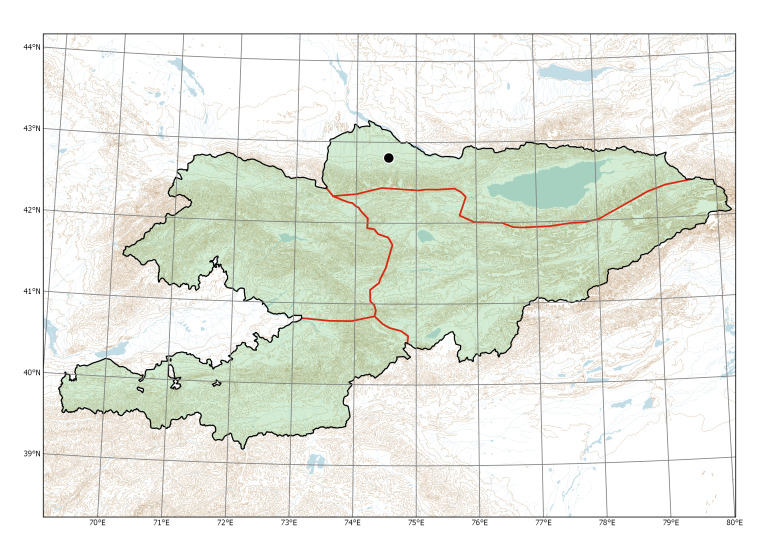
The subspontaneous occurrence of *Daturainnoxia* in Kyrgyzstan.

Northern Tian-Shan (Fig. 6).

So far, the species has been recently recorded from the only locality on the southern margin of Bishkek City, in 2017, by G. Lazkov. A single individual was noticed on a dumping area of the cemetery. This occurrence is apparently casual.

#### Ecology

Dry open forests and shrublands in the native distribution area; cultivated lands, roadsides, ruderal places in the secondary distribution area. It occurs at altitudes of 1200-1800 m a.s.l. in Mexico (Luna-Cavazos and Bye 2011).

#### Biology

Short-lived perennial with a thick root.

#### Notes

The species is a popular ornamental and medicinal plant, also in the native distribution area (Luna-Cavazos and Bye 2011).

#### Introduction to Kyrgyzstan

##### Period of introduction

Neophyte.

**Figure 7.**
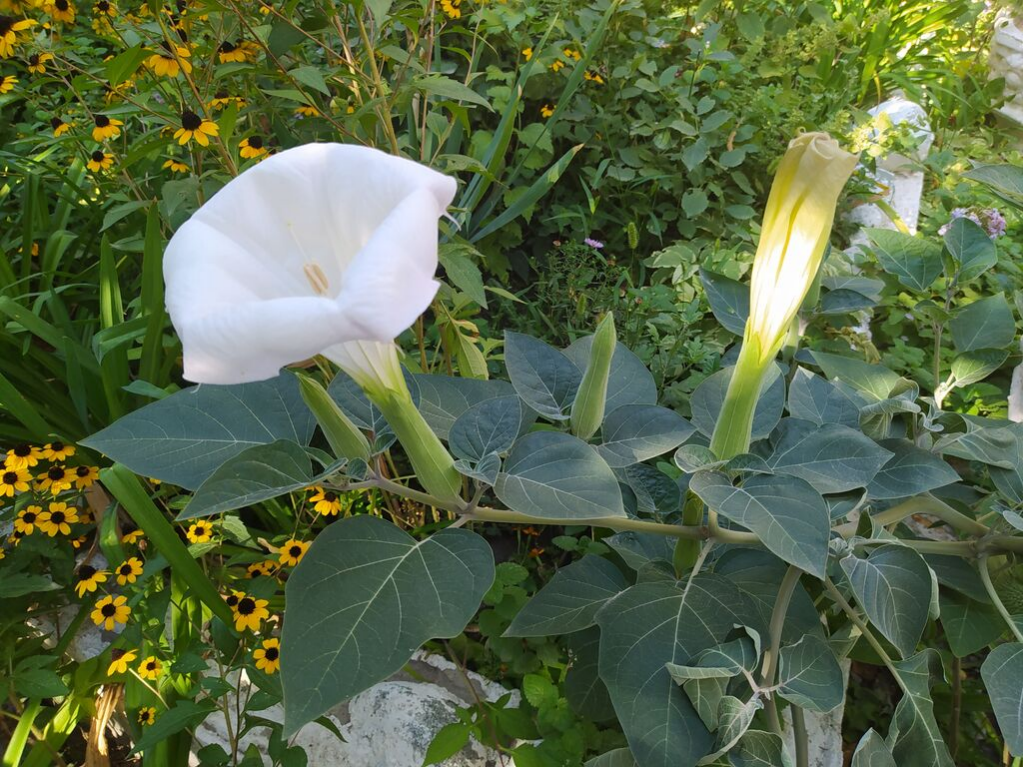
*Daturainnoxia* in ornamental cultivation in Bishkek (photo by G. Lazkov, 21 July 2020).

The species was cultivated in Kyrgyzstan and Uzbekistan for at least 60 years (Nikitina 1960, Kovalevskaya 1961). It was noted to self-seed (Nikitina 1960) but has never been reported as running wild in Kyrgyzstan. Its current subspontaneous occurrence, first recorded in 2017, may be linked with an increasingly common use of the plant in ornamental cultivation, which has been observed in recent years (Lazkov, pers. obs.; Fig. 7).

##### Pathways of introduction

Escape from confinement: Ornamental purpose other than horticulture.

The species is cultivated for ornamental purposes in private gardens and public areas, and is sometimes found in waste sites. As in Europe (e.g. Gudžinskas 2017), its subspontaneous occurrences originated through garden waste. Further dispersal does not occur.

##### Invasion status

Casual.

##### Evidence of impact

Agriculture - no impact (not recorded in crop production areas). Native ecosystems - no impact (restricted to urbanised areas). Urban areas - minor impact (rarely escapes and occurs in ruderal places).

##### Trend

Increasing (inferred).

### Datura stramonium

L. 1753

7422F315-B221-59CD-8772-5BA1F48F4A94

urn:lsid:ipni.org:names:314738-2

Datura stramonium L., Sp. Pl. 1: 179 (1753).
Datura stramonium *Daturatatula*

#### Distribution

##### Native distribution

Central America.

##### Secondary distribution

Archaeophyte in North and South America, Central and Southern Europe, Africa, Southern and Central Asia, Malesia and Australia. Neophyte in Northern Europe. This species is one of the most widely distributed naturalised alien plants in the world, reaching top-10 in the temperate biome (Pyšek et al. 2017).

The species is a striking example of the plants native to the New World but introduced with ancient human-mediated transport in the pre-Columbian era. Plants of *Datura* sp. were introduced to the Old World possibly by the 4th century; the exact mechanism of such transportations is still uncertain (Geeta and Gharaibeh 2007).

##### Distribution in Central Asia

The species occurs as a common alien plant in all the countries of Central Asia (Kovalevskaya 1987).

**Figure 8.**
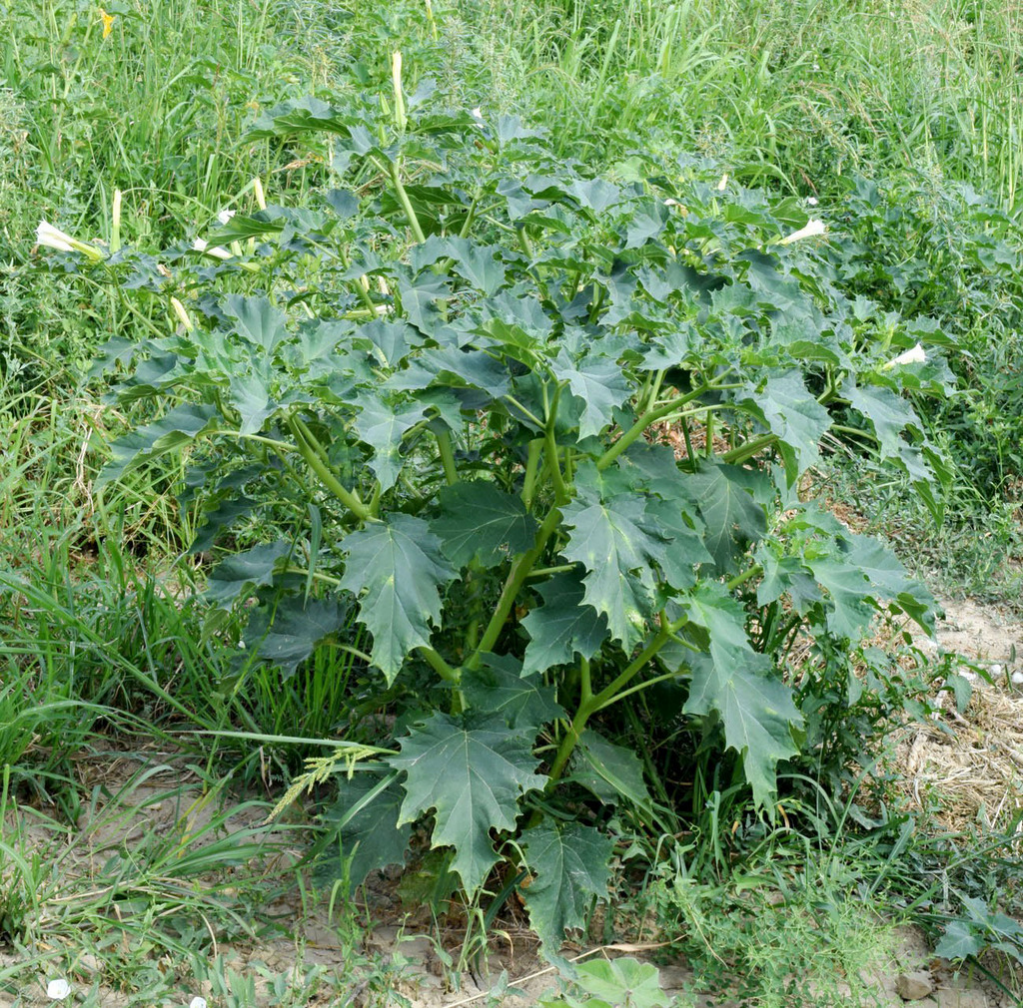
*Daturastramonium* along a cotton field in Andijon District, Uzbekistan (photo by T. Tillaev, 23 July 2017). Source: https://www.plantarium.ru/page/image/id/525187.html (Plantarium 2021).

It was first recorded from Transoxiana (the territories between the Amudarya River and the Syrdarya River) by Avicenna in the first part of the 11th century, although probably as an imported plant (Geeta and Gharaibeh 2007). Nowadays, it is still frequently seen in ruderal places and on field margins (Fig. 8).

##### Distribution in Kyrgyzstan

**Figure 9.**
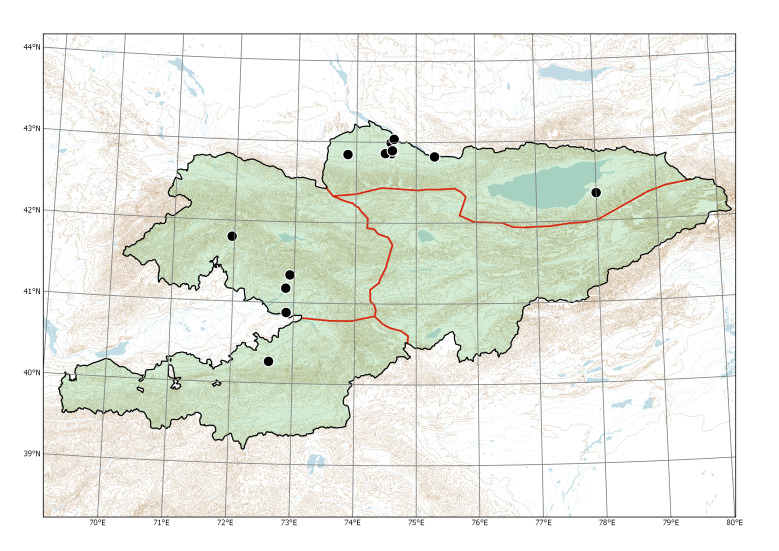
Recorded distribution of *Daturastramonium* in Kyrgyzstan.

Western Tian-Shan, Northern Tian-Shan, Alay-Turkestan (Fig. 9).

The species was found in major agricultural areas (Nikitina 1960) and considered to occur in the whole territory of Kyrgyzstan, although we have not seen collections from the Eastern Tian-Shan. It was collected from populated places and their vicinities, at altitudes up to nearly 2000 m above sea level.

#### Ecology

Dry open forests and shrublands in the native distribution area; cultivated lands, roadsides, ruderal places and riversides in the secondary distribution area. It occurs at altitudes of 500-1200 m a.s.l. in Mexico (Luna-Cavazos and Bye 2011).

In Kyrgyzstan, the species does not occur in high mountains (Nikitina 1960), although Holm et al. (1979) noted its occurrence in the Himalayas as high as 2750 m. Typically, it occurs in or near populated places, in ruderal habitats or along streams in native habitats, sometimes also on fields.

#### Biology

Annual, with a taproot.

#### Taxon discussion

In Central Asia, two species have traditionally been separated, *D.stramonium* with white flowers and *D.tatula* with lilac flowers (Nikitina 1960, Kovalevskaya 1987, Lazkov and Sultanova 2011, Lazkov and Sultanova 2014). This taxonomic distinction is no longer supported (e.g. Safford 1921, Khassanov et al. 2020). Both variants have been found in Kyrgyzstan (Nikitina 1960), although we were not able to find a logical pattern in their distributions.

#### Introduction to Kyrgyzstan

##### Period of introduction

Archaeophyte.

This species was known from the whole of Central Asia from the beginning of its botanical exploration (Fedtschenko and Fedtschenko 1913). The time of its introduction is uncertain, but the species was known from the territory already in the 11th century (Geeta and Gharaibeh 2007). In agreement with the history of the plant introduction described by Geeta and Gharaibeh (2007), we may speculate that the appearance of *Daturastramonium* in Central Asia followed the Muslim reconquest of Transoxiana, which occurred by the beginning of the 11th century and was connected with the massive cultural influence from the Arabic world.

##### Pathways of introduction

Escape from confinement: Horticulture.

The species was originally cultivated as a medicinal plant in India (Geeta and Gharaibeh 2007) and was seemingly introduced as such to Central Asia. Its contemporary occurrence was recorded as a ruderal plant (Deza 1983), with further dispersal by wind and local human activities. Its occasional presence on fields (grain and root vegetables) has also been recorded (Nikitina 1960); in other countries, this may also be a rather new phenomenon, linked with the recent cultivation of soybean, bean and maize that are characterised by larger planting seed material (e.g. Weaver and Warwick 1984).

##### Invasion status

Naturalised. The species is a component of traditional ruderal vegetation, but also occurs along rivers and around springs.

##### Evidence of impact

Agriculture - moderate impact (occasional weed of crops, in fields and gardens). Native ecosystems - minor impact (occurring along streams near populated places). Urban areas - moderate impact (ruderal occurrence).

##### Trend

Stable (inferred).

### Hemerocallis fulva

(L.) L. 1762

F29598F0-705D-5C6E-BB57-C79E65738642

urn:lsid:ipni.org:names:536335-1

Hemerocallis fulva (L.) L., Sp. Pl., ed. 2, 1: 462 (1762) — Hemerocallislilioasphodelusvar.fulva L., Sp. Pl. 1: 324 (1753).

#### Distribution

##### Native distribution

Central and Southern China, Korea, Japan.

##### Secondary distribution

North America, New Zealand (neophyte); Europe, Western and Southern Asia (archaeophyte).

In Europe, the ornamental cultivation of the species has a long history, recorded as common in Britain by Gerard (1597) and in Central Europe by L'Obel (1576) and Clusius (1601) already in the 16th century. It is currently known as naturalised in many countries, including Great Britain (Clement and Foster 1994) and Belgium (Verloove 2021).

*Hemerocallisfulva* was common in the North American ornamental cultivation since the late 19th century; now it became invasive in several states of the USA, occurring along roadsides and river banks (Pennsylvania Flora Database 2021).

##### Distribution in Central Asia

Escaped from traditional ornamental cultivation in Kyrgyzstan and Uzbekistan.

The species was known from subspontaneous occurrences in Kyrgyzstan (Nikitina 1951, Pazij 1971). Its presence in Uzbekistan has not been mentioned in literature, but unpublished herbarium specimens (deposited at LE) were collected from the vicinities of Charvak Village, Tashkent Region (in 1899) and Qora-Qo'rg'on Village, Namangan Region (in 1912).

In historical times, a major part of the mountainous Central Asia, with its highly developed culture in populated oases, was known as Transoxiana (in Latin) or Mavarannahr (in Arabic). This territory, subordinated to various major contemporary states but being *de facto* autonomous, became the Khanate (then Emirate and finally Republic) of Buxoro from the 16th century until 1924. It included two major cities, Buxoro and Samarqand. The Khanate of Buxoro was characterised by extensive cultivation of numerous fruits, vegetables, ornamental and medicinal plants, which were recorded by early European travellers and native writers of Buxoro and Samarqand (e.g. Meyendorff 1826, Abu Tahir Kojo 1899).

The cultivation of *Hemerocallisfulva* in Samarqand was recorded by Olga Fedtschenko in 1869 (Regel 1876). This species, therefore, belongs to the ornamental cultivation of the Khanate of Buxoro, predating the Russian colonisation of the country. Its feral occurrences were recorded in the lower mountains surrounding the Fergana Depression in the early 1870s (Regel 1876), thus indicating that the species was capable of running wild, long before the beginning of the botanical records.

As evident from herbarium records (collections of A. Regel at LE, dated 1877), the species was cultivated also in Qulja [Yining], Xinjiang, China. This means that its historical cultivation apparently included also the agricultural areas of northern Kyrgyzstan.

Since the Khanate of Buxoro maintained close connections and trade of medicinal and other plants with India (Meyendorff 1826), we assume that *Hemerocallisfulva* was originally imported from that country; its broad distribution suggests the early period of introduction. It is also possible that the species was first imported as a vegetable, for its edible flowers and fleshy rhizomes (Li 1970), and was subsequently turned into an ornamental.

**Figure 10.**
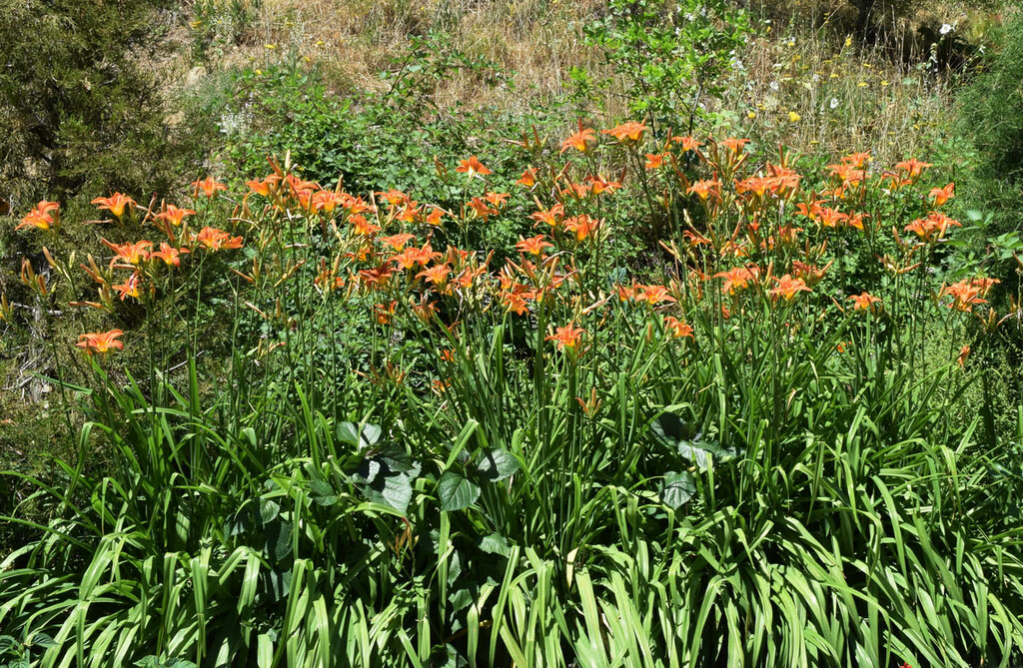
*Hemerocallisfulva* in modern ornamental cultivation in Tashkent Region, Uzbekistan (photo by T. Tillaev, 28 June 2018). Source: https://www.plantarium.ru/page/image/id/582460.html (Plantarium 2021).

Currently, the species is very commonly cultivated in Central Asia (Fig. 10), although seemingly from some commercial sources, different from the historical cultivation. Its recent subspontaneous populations are not recorded.

##### Distribution in Kyrgyzstan

**Figure 11.**
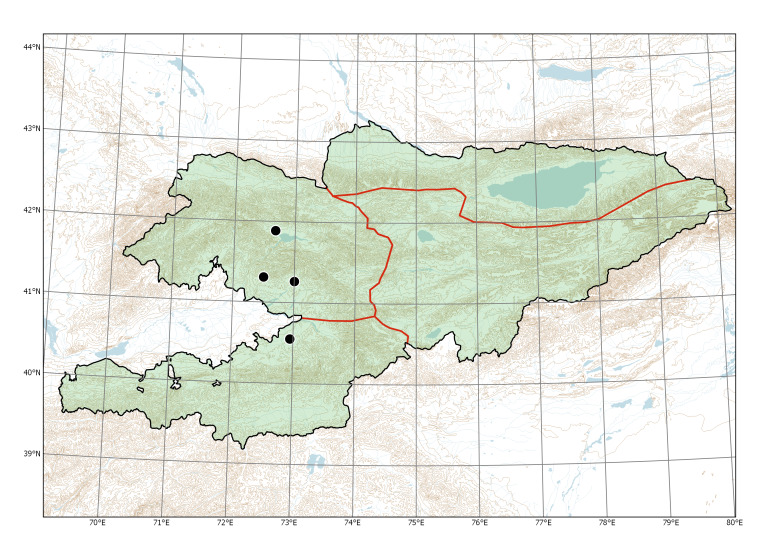
Historical records of *Hemerocallisfulva* in Kyrgyzstan.

Western Tian-Shan, Alay-Turkestan (Fig. 11).

The species was found along rivers and irrigation ditches in the lower mountain belt (950-1100 m a.s.l.) near populated places surrounding the Fergana Depression.

#### Ecology

Riversides in forests and grasslands in the native distribution area; stream sides, road sides and grasslands in the secondary distribution area.

In China, the species was recorded at altitudes of 300-2500 m (Chen and Noguchi 2000). In the secondary distribution area, it was recorded in the Indian Himalayas as high as 1600-2200 m above sea level (Khuroo et al. 2006), whereas in the Caucasus it occurred mostly at lower altitudes (Grossheim 1940). According to herbarium specimens, the historical localities of *Hemerocallisfulva* in Central Asia were situated at 700-1100 m above sea level.

#### Biology

Rhizomatous perennial. Flowers opening diurnal half-day, due to specialisation to diurnal moths (Hirota et al. 2021). Easily propagated by rhizomes, resulting in monoclonal cultivation (Stout 1921). Plants in cultivation are largely sterile, with undeveloped seed capsules (Grier 1914), which is explained by their triploid chromosome number (Stout 1932). Such triploid clones may naturally occur in the wild (Matsuoka 1971).

#### Notes

According to the specimens examined, the traditional cultivation in Central Asia was represented by at least two forms; one was slender with narrow leaves and the other was more robust. The fruits were not developed, thus indicating triploidy. Double-flowered forms were not observed.

#### Introduction to Kyrgyzstan

##### Period of introduction

Archaeophyte.

The species was common in ornamental cultivation in the Khanate of Buxoro, and found in the territories around the Fergana Depression that belonged to this state. This introduction is at least some centuries old.

##### Pathways of introduction

Escape from confinement: Ornamental purpose other than horticulture.

Although the plant is edible, its latest historical use was ornamental cultivation in private gardens (Regel 1876).

##### Invasion status

Locally naturalised, maintained by vegetative reproduction (colonophyte).

**Figure 12.**
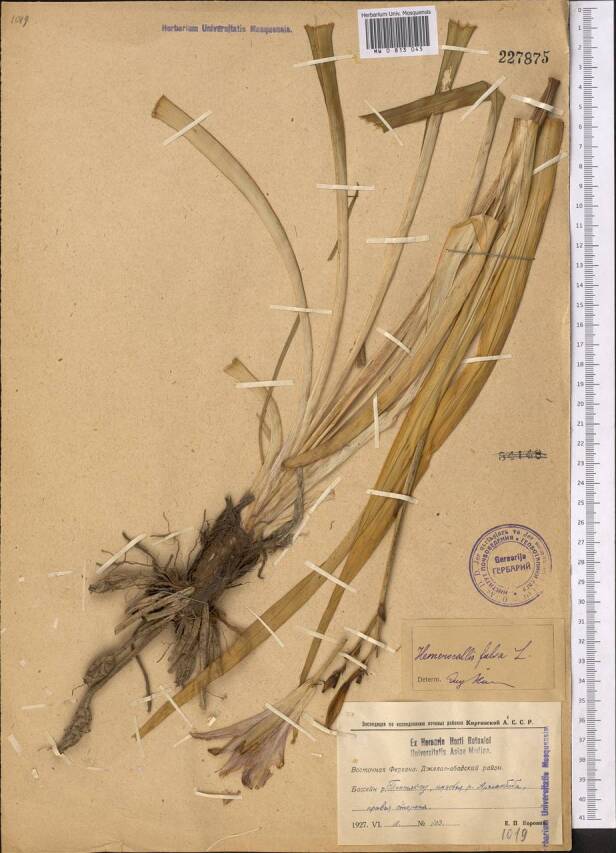
The last historical specimen of *Hemerocallisfulva* from Kyrgyzstan (MW0813045).

In Kyrgyzstan, feral populations of the species were known along rivers and irrigation ditches near populated places, from the area of semi-wild apple and walnut forests at the lower belt in the north-western part of the Fergana Range (Pazij 1971). These populations had been repeatedly sampled from the early 1870s till 1927 (Fig. 12), thus indicating their conspicuousness. Although these territories belong to the most visited and intensely studied areas in the country (e.g. Sukachev 1949), no further collections or observations originated in the latest 95 years; this indicates that the populations had significantly declined or even disappeared. Their current status or even existence have not been verified; the old feral populations may be currently extinct.

##### Evidence of impact

Agriculture - no impact (not weedy). Native ecosystems - minor impact (colonising riversides near populated places). Urban areas - minor impact (colonising irrigation ditches in populated places).

##### Trend

Declining (inferred).

### Hyoscyamus niger

L. 1753

8A8F7537-B0AD-5DC5-9DD6-23D1F7DEF7A2

urn:lsid:ipni.org:names:815932-1

Hyoscyamus niger L., Sp. Pl. 1: 179 (1753).

#### Distribution

##### Native distribution

Mediterranean, Western Asia (Meusel and Jäger 1965).

##### Secondary distribution

Archaeophyte in Temperate and Northern Europe and Temperate Asia, neophyte in Australia and North America.

##### Distribution in Central Asia

The species occurs in all the countries of Central Asia (Kovalevskaya 1987).

##### Distribution in Kyrgyzstan

**Figure 13.**
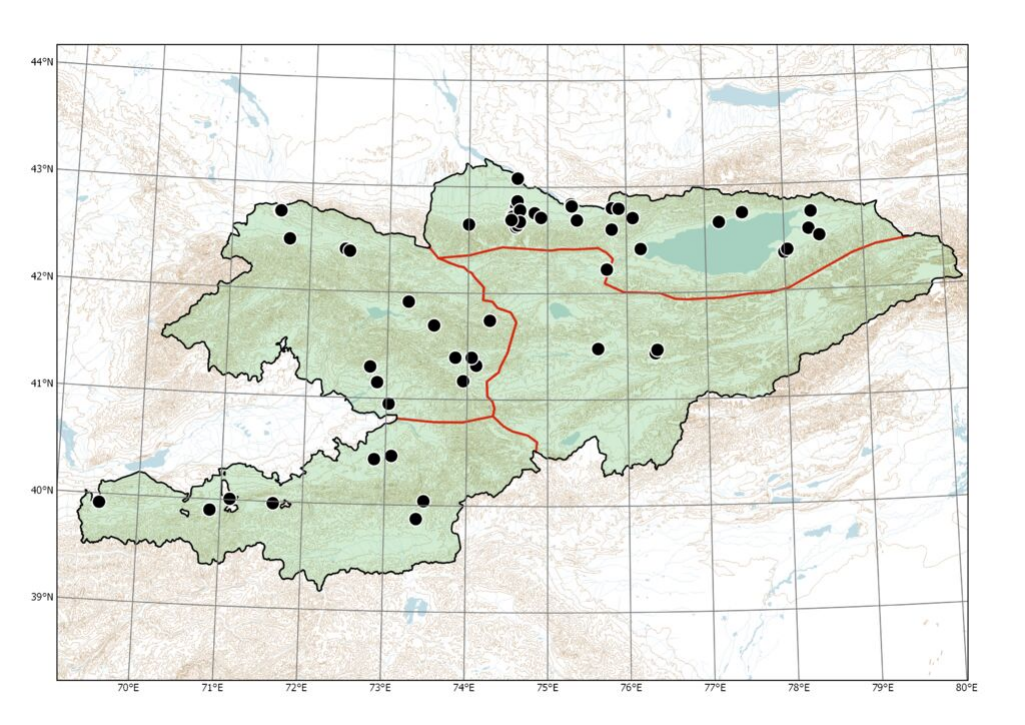
Recorded distribution of *Hyoscyamusniger* in Kyrgyzstan.

Western Tian-Shan, Northern Tian-Shan, Eastern Tian-Shan, Alay-Turkestan (Fig. 13).

The species is distributed in agricultural areas and populated places across the whole territory of Kyrgyzstan (Nikitina 1960, Deza 1983).

#### Ecology

Stony or rocky places in the native distribution area, roadsides, fields, yards, waste places in the secondary distribution area.

In Kyrgyzstan, the species was recorded as occurring in agricultural areas up to the lower mountain belt (Nikitina 1960), but its ruderal occurrence may reach the elevations as high as 2600 m according to the specimens examined; its field occurrence was probably common at elevations up to 2000 m. In the Caucasus, *Hyoscyamusniger* may reach the upper mountain belt (Grossheim 1967).

#### Biology

Annual or more commonly biennial, with a taproot.

#### Notes

This species is a traditional medicinal plant, used since ancient times in the Roman Empire (Mitich 1992) and is still an official drug in some countries, like the UK or used to obtain alkaloids (Hocking 1947, Morton 1977), for which the plant has been commercially cultivated (Mitich 1992). It was popular in the Middle Ages in Europe, reaching as far north as Finland with medieval cultivation (Lempiäinen 1991). It is a traditional medicinal plant in Iran (Moattar and Moattar 2004) and China (Xiong et al. 2018); it is also used in the Indian *Ayurveda* (Aparna et al. 2015). In India, the species was commonly used in local medicine in the 19th century (Royle 1839); in Europe, it remained in use at least before the Second World War (Perkamaitė and Gudienė 2015).

The closest relative of *Hyoscyamusniger* is *H.albus* L., which also occurs as native in the Mediterranean and Western Asia (Sanchez-Puerta and Abbona 2014).

#### Introduction to Kyrgyzstan

##### Period of introduction

Archaeophyte.

The species was first recorded as being in foreign use in medieval China, Tang Dynasty (Li 1977, Li 2012), corresponding to the 7th-9th centuries. This can be firmly linked with the Muslim conquest of Transoxiana, which occurred during AD 673-751 (Nicolle 2009). Besides the narcotic effect, seeds of the plant were used as a tonic that provides strength in walking for long distances (Li 1977, Li 2012); this effect apparently was valued in the contemporary army.

##### Pathways of introduction

Escape from confinement: Horticulture.

As the plant is an important sedative, anaesthetic and pain-relieving drug of ancient times, we conclude that it was intentionally introduced with medical purposes and subsequently cultivated in Central Asia. This ancient cultivation has been abandoned long ago, and the species largely occurs as a ruderal plant in or around populated places, or as a weed, or on abandoned fields (Nikitina 1960, Kovalevskaya 1987). Many researchers (Nikitina 1960, Deza 1983, Kovalevskaya 1987) also noted its common occurrence on fields, which assumes its secondary dispersal with contaminated seeds and from ruderal habitats. Its ruderal occurrence relies on winds and human activities.

##### Invasion status

**Figure 14.**
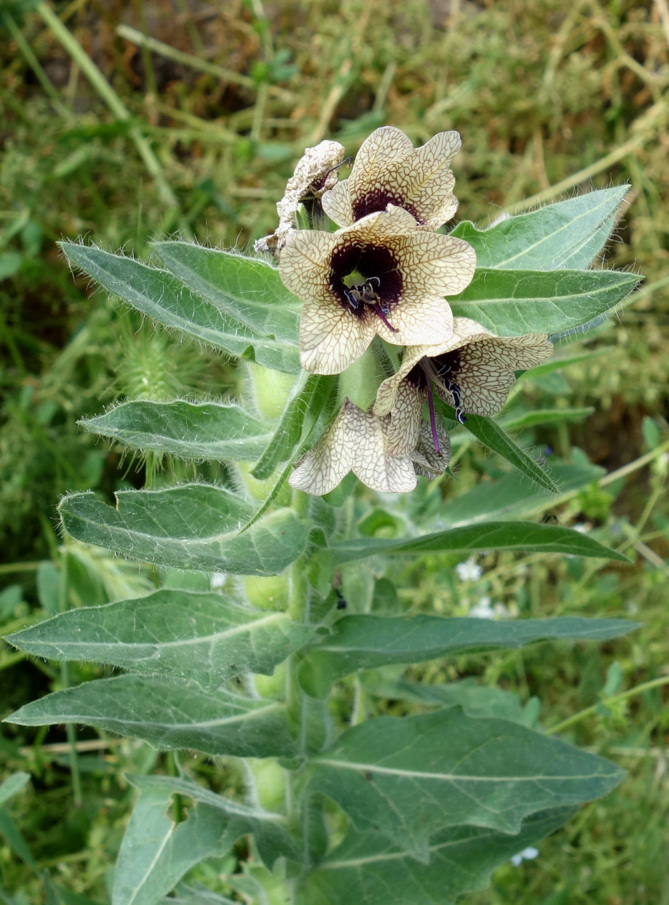
*Hyoscyamusniger* near Bishkek City, Kyrgyzstan (photo by G. Chulanova, 25 May 2019). Source: https://www.plantarium.ru/page/image/id/645576.html (Plantarium 2021).

Naturalised, old invasive plant. The species has been very frequently found in populated places (ruderal places, roadsides) and on fields (wheat, alfalfa) and pastures (Nikitina 1960, Kovalevskaya 1987), but may also occur in native habitats (along riversides, on rocky slopes, in grasslands) near populated places. It is still regularly found in the country (Fig. 14).

##### Evidence of impact

Agriculture - major impact (reported as a common weed of crops, on fields and in gardens). Native ecosystems - major impact (occurring in natural habitats near populated places). Urban areas - major impact (ruderal occurrence).

##### Trend

Stable (inferred).

### Nicandra physalodes

(L.) Gaertn. 1791

FD5646C1-F347-5162-BBD0-843302B6AEE6

urn:lsid:ipni.org:names:816832-1

Nicandra physalodes (L.) Gaertn., Fruct. Sem. Pl. 2: 237 (1791) — *Atropaphysalodes* L., Sp. Pl. 1: 181 (1753).

#### Distribution

##### Native distribution

South America.

##### Secondary distribution

North and Central America, Southern Europe and Asia, Africa, Australia.

In Europe, this species belongs to the most widely distributed alien vascular plants (Lambdon et al. 2008).

The species has been naturalised in several provinces of China (Zhang et al. 1994), probably as a field weed.

##### Distribution in Central Asia

Sporadically found in Kazakhstan, Kyrgyzstan, Turkmenistan, Uzbekistan (Kovalevskaya 1987).

In Central Asia, the species was historically found in gardens and on melon fields (Pojarkova 1954b, Nikitina 1960, Vasilieva 1965). In the early 20th century (1907-1931), it was recorded as cultivated and ruderal in vegetable gardens of Russian-populated places.

The first record of the species from Central Asia comes from Vannovskoe Village of Turkestan Region (now Tūrar Rysqūlov Village, Türkıstan Region, Kazakhstan). This village was founded by Russian and Ukrainian colonists in 1887, which may be close to the earliest possible date of the species' introduction to Central Asia (following the conquest in 1868).

Its recent occurrence seems to be in ornamental cultivation and ruderal.

##### Distribution in Kyrgyzstan

**Figure 15.**
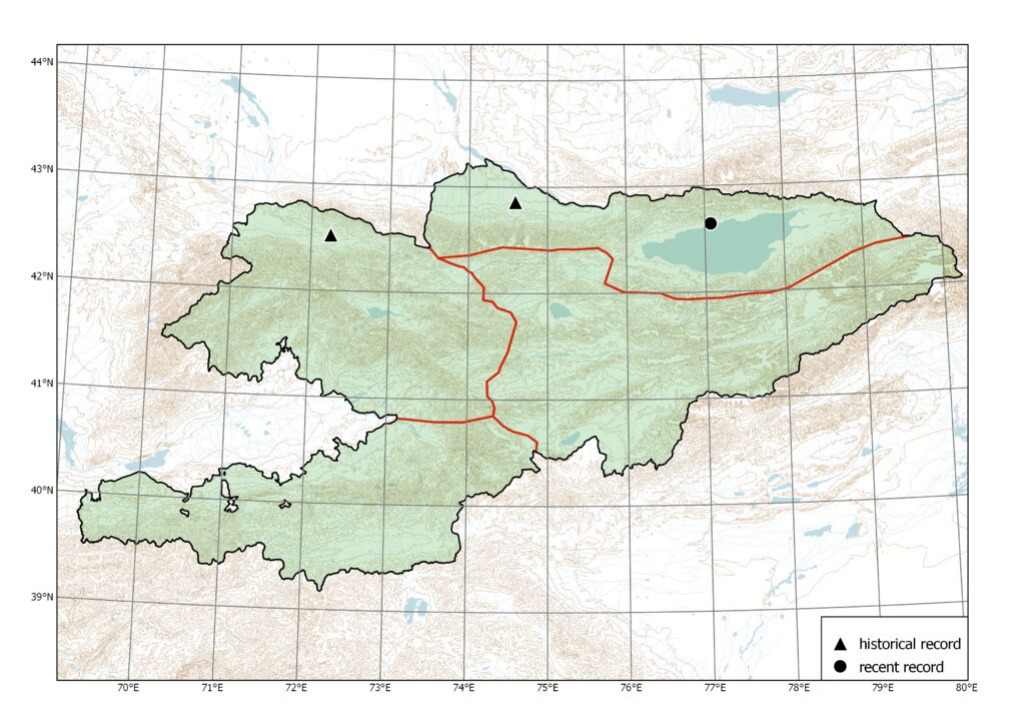
Historical and recent records of *Nicandraphysalodes* in Kyrgyzstan.

Western Tian-Shan, Northern Tian-Shan (Fig. 15).

**Figure 16.**
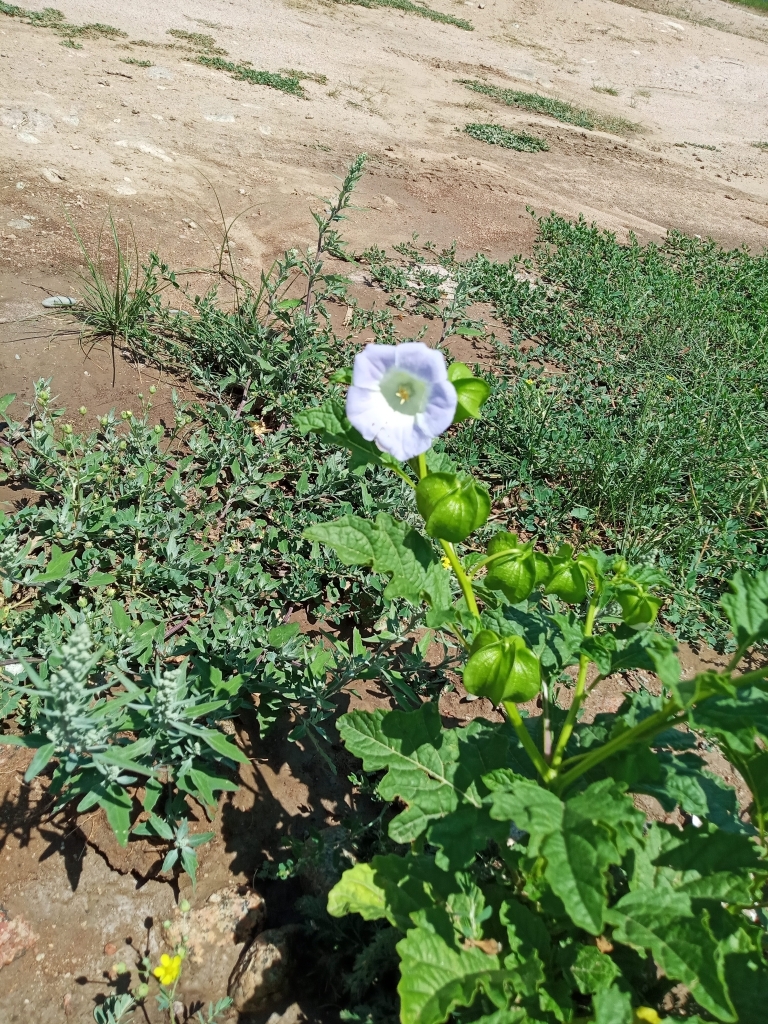
*Nicandraphysalodes* in Cholpon-Ata, Kyrgyzstan (photographed 25 July 2020). Source: https://www.inaturalist.org/observations/54240496 (iNaturalist 2021).

The species was found as a weed in agricultural areas of the Chü and Talas Depressions (Nikitina 1960). Recently, it was rediscovered as a ruderal plant (Fig. 16) in a village along the northern side of Ysyk-Köl Lake (iNaturalist 2021).

#### Ecology

Fertile places in the native distribution area; ruderal places, gardens, fields and field margins, roadsides and pastures in the secondary distribution area.

#### Biology

Annual, with a taproot.

#### Notes

Historically, e.g. in the 1920s, *Nicandraphysalodes* was rather commonly used as a surrogate for *Humuluslupulus* in bakery in southern Russian and Ukrainian villages (Larionov 1931). Due to this use, the plants were spread across a vast territory and became locally abundant in village gardens in Siberia (Khrebtov 1926) and Central Asia (Cherniakovskaya 1935). This usage has been abandoned, and the plants introduced in those times have subsequently disappeared.

#### Introduction to Kyrgyzstan

##### Period of introduction

Neophyte.

The plant was introduced in the early Soviet period (first record in 1928 from present-day Talas Town), transported by Russian colonists from their native villages in southern Russia. Its latest record from the Botanical Garden in Bishkek is dated 1955 and may constitute the last remnant of the old cultivation.

Its latest subspontaneous record is dated 2020 and may be linked with recent ornamental cultivation.

##### Pathways of introduction

Escape from confinement: Agriculture. Escape from confinement: Ornamental purpose other than horticulture.

The plant seems to have been historically introduced for its cultivation and subsequent use in home bakery, and then it has become a weed in and around the places of introduction. This pathway is indicated by the contemporary evidence (Khrebtov 1926, Larionov 1931) and by the recorded occurrences of the plants in vegetable gardens.

Modern pathways of introduction of this plant in Europe include grain import (Suominen 1979, Clement and Foster 1994, Verloove 2021), wool contamination and horticulture (Clement and Foster 1994, Verloove 2021). The species is a very common weed of many crops, especially in warmer countries (Holm et al. 1997).

In Russia, in recent years, *Nicandraphysalodes* has been widely cultivated for ornamental purposes in private gardens and city yards and along streets, and occasionally noted as running wild and occurring in ruderal and dumping places (Plantarium 2021).

A recent record from Ysyk-Köl Region, Kyrgyzstan (iNaturalist 2021) indicated that the species may escape from ornamental cultivation and occur in ruderal places as a casual alien.

Further dispersal was not observed.

##### Invasion status

Casual.

The historical occurrences have seemingly disappeared (the plant was not naturalised and the historical factors of introduction are no longer in place). The recent ruderal occurrence was represented by a single plant and was apparently casual as well.

##### Evidence of impact

Agriculture - no impact (no longer occurring as a weed in gardens or on fields). Native ecosystems - no impact (not found outside populated places). Urban areas - minor impact (ruderal occurrences).

##### Trend

Increasing (inferred).

### Physalis angulata

L. 1753

5C6EBC2E-B91E-5B10-A86B-B5F787980E76

urn:lsid:ipni.org:names:195334-2

Physalis angulata L., Sp. Pl. 1: 183 (1753).
Physalis angulata *Physalisminima*
Physalis angulata *Physalishermannii*

#### Diagnosis

**Figure 17.**
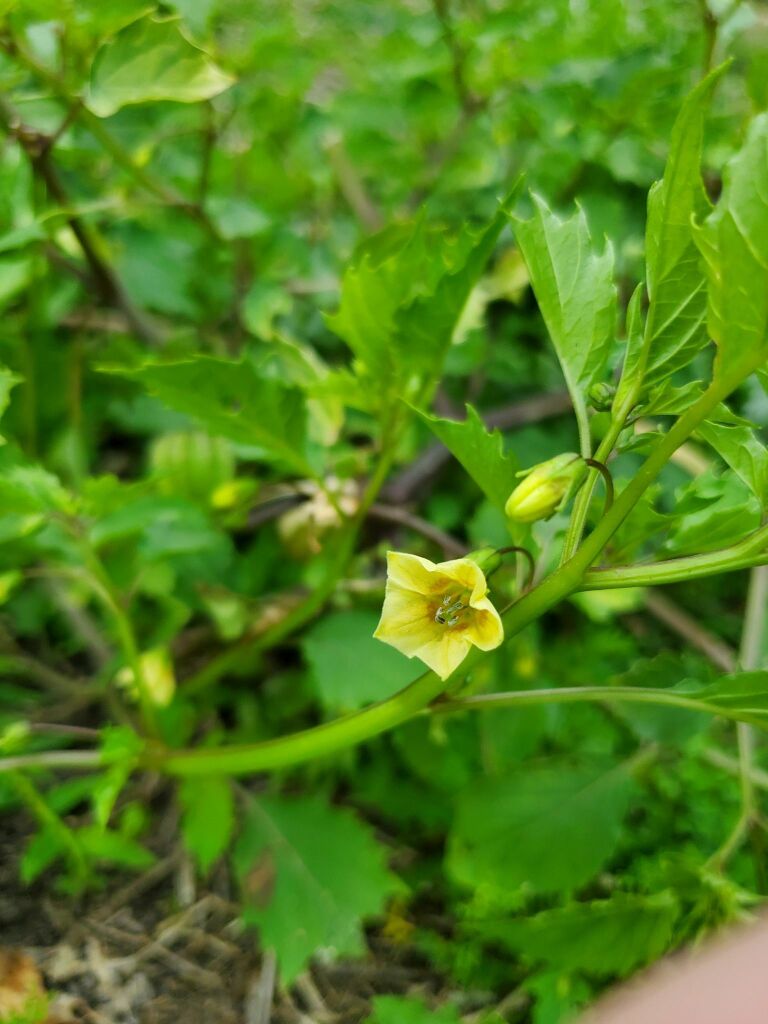
Leaves and flowers of *Physalisangulata* (Louisiana, USA, 2021). Source: https://www.inaturalist.org/photos/111793511 (iNaturalist 2021).

**Figure 18.**
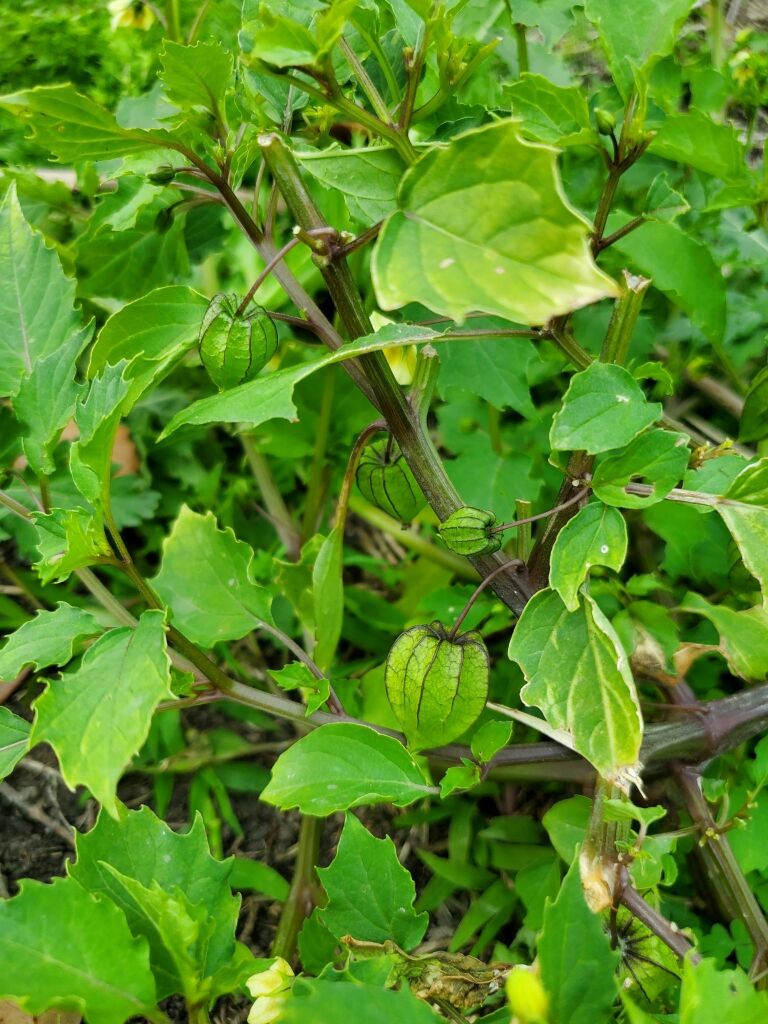
Fruits of *Physalisangulata* (Louisiana, USA, 2021). Source: https://www.inaturalist.org/photos/111793520 (iNaturalist 2021).

Among the annual species of *Physalis* occurring as aliens in Central Asia, *P.angulata* was sometimes confused with *P.philadelphica*. It differs from the latter by ovate-elliptic leaves on longer petioles, pale yellow or whitish-yellow corollae with small pale brown spots at the base (Fig. 17), longer pedicels, and fruiting calyces prominently angled in fruit (Fig. 18) (Terrones et al. 2020).

#### Distribution

##### Native distribution

Central and South America.

##### Secondary distribution

Africa, Europe, southern Asia, Australia, North America.

##### Distribution in Central Asia

Tajikistan, Uzbekistan. Previously reported in error from Kyrgyzstan.

In Tajikistan, the species was frequently found during the period between 1928 and the 1960s on cotton and sesame fields in the large oasis of Boxtar (Korovin 1934, Kovalevskaya 1986), which is a large agricultural centre of the southern part of the country. Recently it was sporadically noted on rice fields (Nowak et al. 2013).

**Figure 19.**
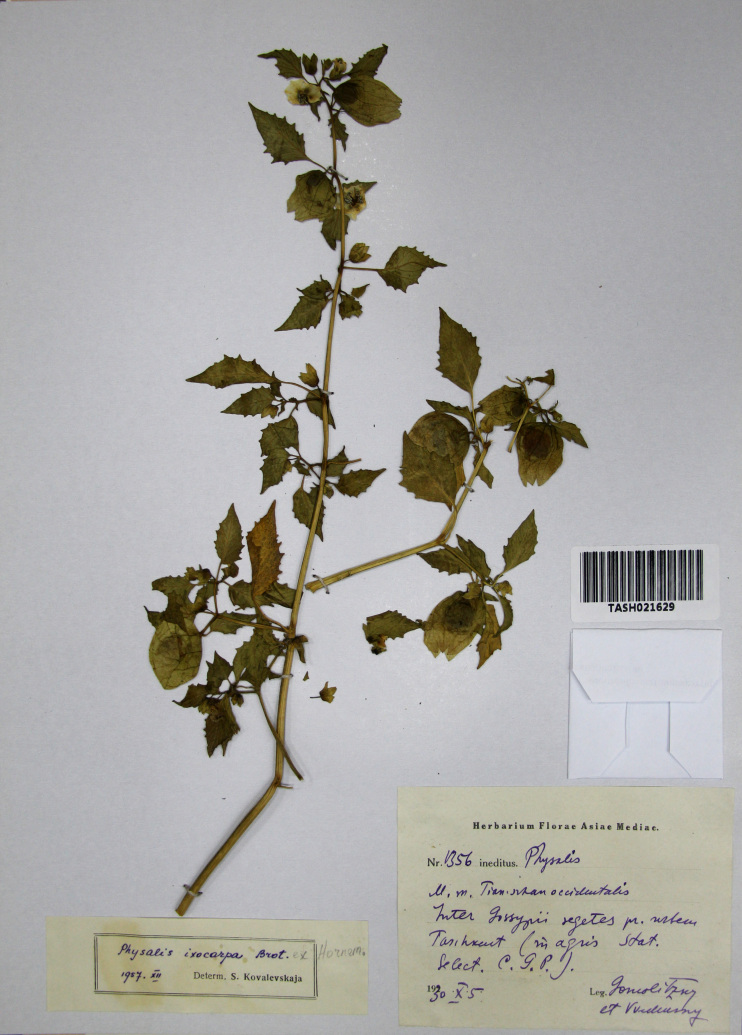
A specimen of *Physalisangulata* from Uzbekistan, which was misidentified as *P.ixocarpa* (TASH).

During the same period, it was recorded on cotton fields also in Uzbekistan, near Tashkent and Samarkand (Kovalevskaya 1961). Some older specimens, collected on cotton fields near Tashkent, were misidentified by Kovalevskaya (1961) as *Physalisixocarpa*. These specimens (Fig. 19) are characterised by pale whitish flowers with very faint spots in the throat and by long pedicels, thus matching *P.angulata*. For this reason, the historical record of *P.ixocarpa* from Uzbekistan (Khassanov et al. 2020) should be rejected.

The only historical record of *P.angulata* from Bishkek, Kyrgyzstan (Kovalevskaya 1987) is based on herbarium specimens collected from experimental cultivation and kept at FRU and LE. On the basis of this record, the species was listed as occurring in the country (Lazkov and Sultanova 2011, Lazkov and Sultanova 2014). Since this occurrence was not spontaneous, the species should be removed from the flora of Kyrgyzstan.

It seems that *P.angulata* was introduced to Central Asia largely with cotton cultivation (American varieties introduced in the late 1920s). According to herbarium records, the species persisted on and around cotton fields until the 1960s. No recent records are available, and the current status of the species is unknown (presumably historical casual).

##### Distribution in Kyrgyzstan

No spontaneous occurrence has been recorded.

#### Ecology

Probably open riversides in the native distribution area; riversides, roadsides, fields and fields margins, ruderal places in the secondary distribution area.

#### Biology

Annual, with a taproot.

#### Notes

Small-flowered variants of *Physalisangulata* were reported from Central Asia as *P.minima* (Korovin 1934, Cherniakovskaya 1935) or *P.hermannii* (Kovalevskaya 1961, Kovalevskaya 1986, Kovalevskaya 1987). Such plants do not deserve taxonomic recognition at any rank.

### Physalis philadelphica

Lam. 1786

D12B287C-9C75-5262-B18B-DD7702FE4EEE

urn:lsid:ipni.org:names:817532-1

Physalis philadelphica Lam., Encycl. 2(1): 101 (1786).
Physalis philadelphica *Physalisixocarpa*Physalisphiladelphicasubsp.ixocarpa

#### Diagnosis

**Figure 20.**
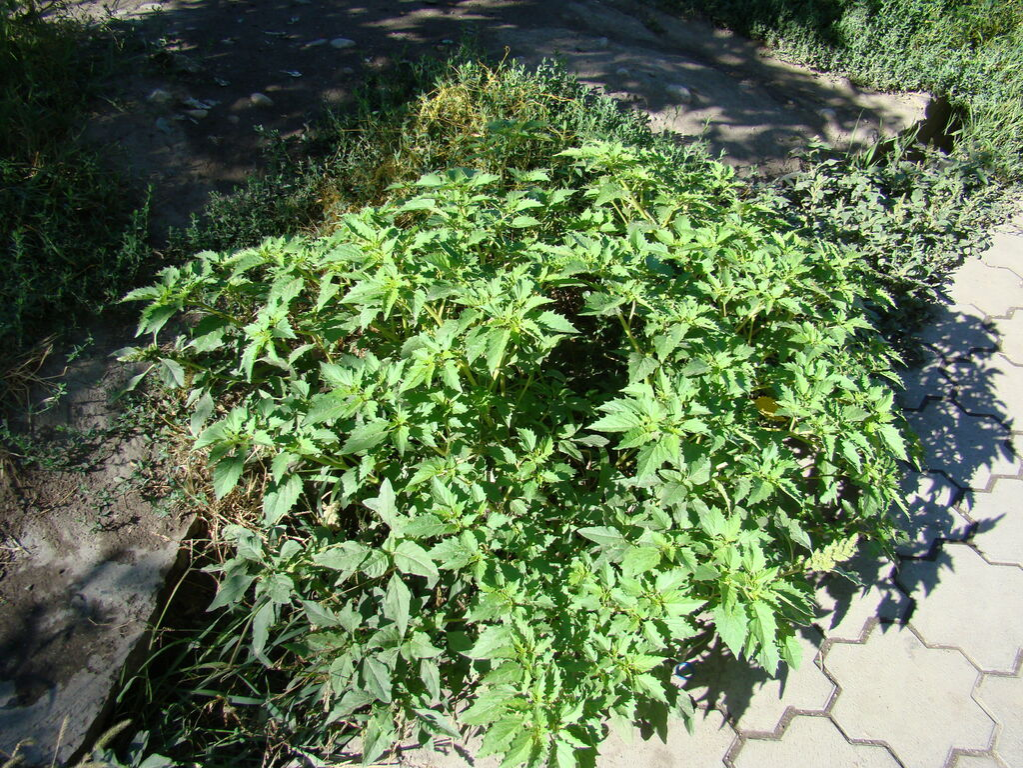
*Physalisphiladelphica* in Bishkek (single plant) (photo by G. Lazkov, 15 August 2015).

**Figure 21.**
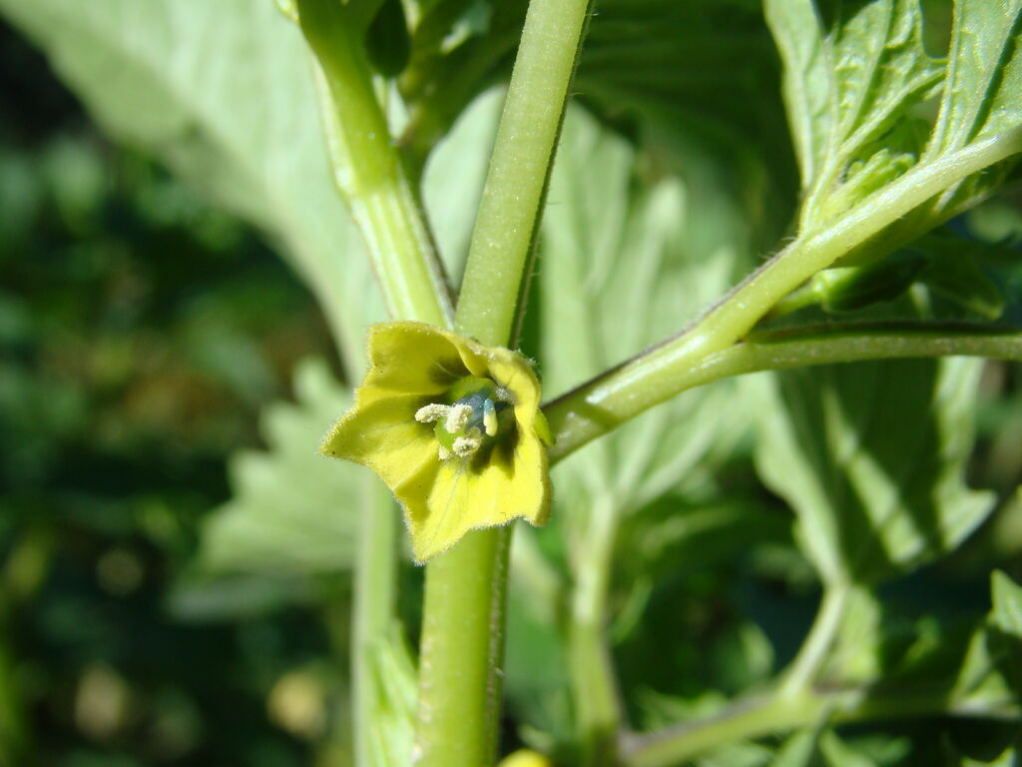
*Physalisphiladelphica* in Bishkek (small-sized flower) (photo by G. Lazkov, 15 August 2015).

**Figure 22.**
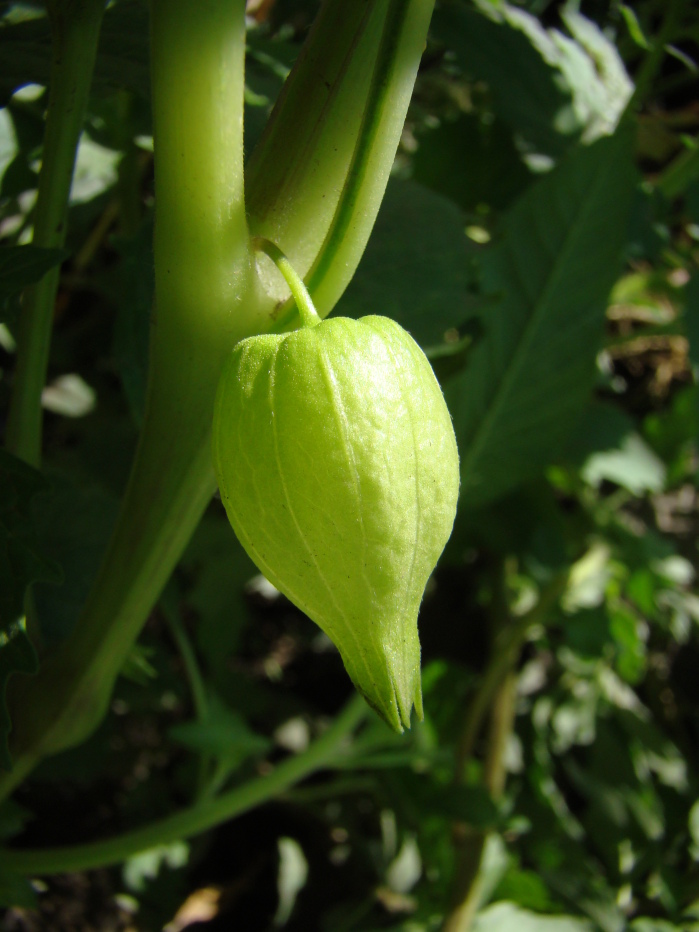
*Physalisphiladelphica* in Bishkek (immature fruits) (photo by G. Lazkov, 15 August 2015).

In Central Asia, *P.philadelphica* has been commonly confused with *P.angulata*. It differs from the latter by ovate-lanceolate leaves on shorter petioles (Fig. 20), intensely yellow corollae with prominent dark brown spots at the base (Fig. 21), shorter pedicels, and fruiting calyces indistinctly (Fig. 22) angled in fruit (Terrones et al. 2020).

#### Distribution

##### Native distribution

Native to Central America.

##### Secondary distribution

Widely cultivated as a fruit crop. Introduced in many countries of North America, Europe, Africa, Asia, Australia. Archaeophyte in North America (González-Pérez and Guerrero-Beltrán 2021), neophyte elsewhere.

In arid areas of Asia, the species was introduced to Turkey in the 1990s as a weed of irrigated cotton fields (Bükün et al. 2002), where it became invasive and quickly gained the top status among other weeds (Bükün 2004). In other arid territories, this species is not invasive.

##### Distribution in Central Asia

Reported from Kazakhstan, Kyrgyzstan and Uzbekistan for the first time here.

In Kazakhstan, the species was observed for the first (and only) time in 2019, in a damp place at Jabağyly Village, Turkestan Region (Plantarium 2021).

In Kyrgyzstan, it was found for the first time in Bishkek, in 2015.

In Uzbekistan, the species was originally recorded in 1929 and 1930 as a weed of cotton cultivation (Kovalevskaya 1961, Khassanov et al. 2020), but this record was erroneously based on misidentified specimens of *P.angulata* (see discussion under that species). Since 2008, the species was repeatedly collected as a ruderal plant in Tashkent (Plantarium 2021). Besides, recently it was noted (albeit with a low abundance) as a weed in Tashkent Region: on late carrot fields in Oqqoʻrgʻon District (Axmedov and Narbaev 2019), on tomato fields in Qibray District (Axmedov et al. 2020) and along margins of maize fields in Qibray District (Plantarium 2021). All recent records were misidentified as *P.angulata*.

##### Distribution in Kyrgyzstan

**Figure 23.**
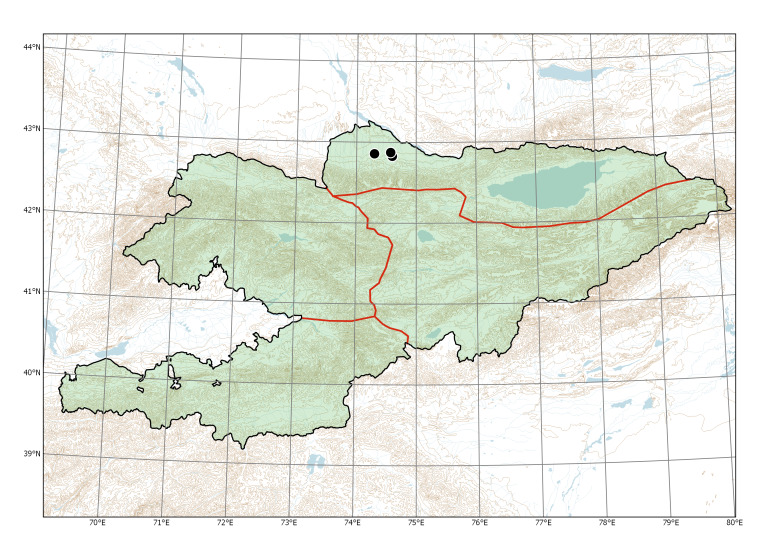
Distribution of *Physalisphiladelphica* in Kyrgyzstan.

Northern Tian-Shan (Fig. 23).

The species was first recorded by Georgy Lazkov in Bishkek City in 2015, as a few scattered individuals along Toktogul Street and a large population (ca. 20 flowering individuals) on the southern margin of the city. The scattered individuals withered quickly, whereas the fate of the large population was not studied. Further on, one individual was observed in flower in Sokuluk Village in 2021. The plants usually occurred in places with regular water supply, along irrigation ditches.

#### Ecology

Open or partly shaded places on humid, fertile soils in the native distribution area; roadsides, fields, field margins, ditches and riversides, dumps and ruderal places in the secondary distribution area.

In arid areas, the species depends on the availability of water supply (Bükün et al. 2002).

#### Biology

Annual or short-lived perennial (Rydberg 1896), with a taproot.

Plants of *Physalisphiladelphica* are self-incompatible (Mulato-Brito et al. 2007). This genetic feature reduces the chances for reproduction of single plants or small colonies derived from a single source of introduction.

#### Notes

*Physalisixocarpa* is sometimes separated from *P.philadelphica* s. str., at the rank of species or subspecies (e.g. Rydberg 1896, Stace 2010). The alleged differences are in the size of corolla (5-10 mm in diam. in *P.ixocarpa*, 10-25 mm in diam. in *P.philadelphica*), with corresponding differences in the size of calyx and fruit. These dimensional characters match the infraspecific variability observed in Mexico (Zamora-Tavares et al. 2014), part of the native distribution area of *P.philadelphica*, thus evidencing that the two putative taxa are synonyms. The taxonomy of this species is still unsettled, with some authors being reluctant to accept a broader concept (e.g. Pretz and Deanna 2020).

The plants observed in Kyrgyzstan were small-flowered, thus corresponding to *P.ixocarpa*.

*Physalisphiladelphica* has been an important crop in Mexico since pre-Columbian times, and now it is cultivated globally as 'tomatillo' for its edible fruits (Small 2011). The long history of cultivation, as well as the presence of wild, cultivated and weedy populations already in the country of origin, may account for its high level of morphological variability (Zamora-Tavares et al. 2014).

#### Introduction to Kyrgyzstan

##### Period of introduction

Neophyte.

The first record from Kyrgyzstan was dated 2015, thus falling within the period of the independence.

##### Pathways of introduction

Transport - Contaminant: Seed contaminant.

In Kyrgyzstan, the species is sometimes cultivated in private gardens and sold privately in marketplaces (Lazkov, pers. obs.), but its direct escape from cultivation is considered highly unlikely. Its occurrences on roadsides and waste ground in populated places suggest the arrival with contaminated grain or fodder. No further dispersal was noticed.

In Uzbekistan, the species with certainly arrived with contaminated seed material, as indicated by its occurrence on fields. A wide variety of contaminated seed material (carrots, tomato, maize) indicates its North American origin and multiple sources of introduction.

In Russia, the species was introduced in the European part with garden seeds, as a weed of flower beds and vegetable gardens, recorded in the Middle Volga Region in the 1990s (Rakov et al. 2011). Its earliest record in Siberia (Novosibirsk, dated 1944) comes from potato fields (Ebel et al. 2015).

In the USA, the species is cultivated for fruits and frequently escapes from cultivation, becoming established along roadsides and field margins (Sullivan 2004). In Kenya, its original introduction was intentional as a green manure crop that caused a local invasion (Cunningham-van Someren 1957). In the British Isles, the species originated with grain, wool and food refuse (Clement and Foster 1994); it was also known as a contaminant of bird seed (Hanson and Mason 1985).

Further dispersal in other countries was registered as occurring with animals (Cunningham-van Someren 1957) and water (Bükün et al. 2002). Its riverbed occurrence in Spain (Gómez-Bellver et al. 2016) seems to be connected with this type of dispersal.

##### Source of introduction

Presumably North America.

##### Invasion status

Casual; ephemeral or locally persisting. The species may become established in places with regular water supply; so far, no long-term survival has been observed.

##### Evidence of impact

Agriculture - no impact (so far, not recorded on fields, although recent surveys are lacking). Native ecosystems - no impact (not found outside populated places). Urban areas - minor impact (casual occurrence as a ruderal plant).

##### Trend

Increasing (observed).

The species has been noticed in Kyrgyzstan only recently, as a newcomer. Its regular recent occurrence as a weed or ruderal plant in Uzbekistan may suggest further spreading also in Kyrgyzstan. Its recent introduction and subsequent expansion in Turkey (Economou et al. 2016) indicates that the species is potentially invasive in regularly irrigated areas.

### Solanum nigrum

L. 1753

8488BF57-4338-5B34-84F4-202C9744AE36

urn:lsid:ipni.org:names:30048260-2

Solanum nigrum L., Sp. Pl. 1: 186 (1753).

#### Diagnosis

**Figure 24.**
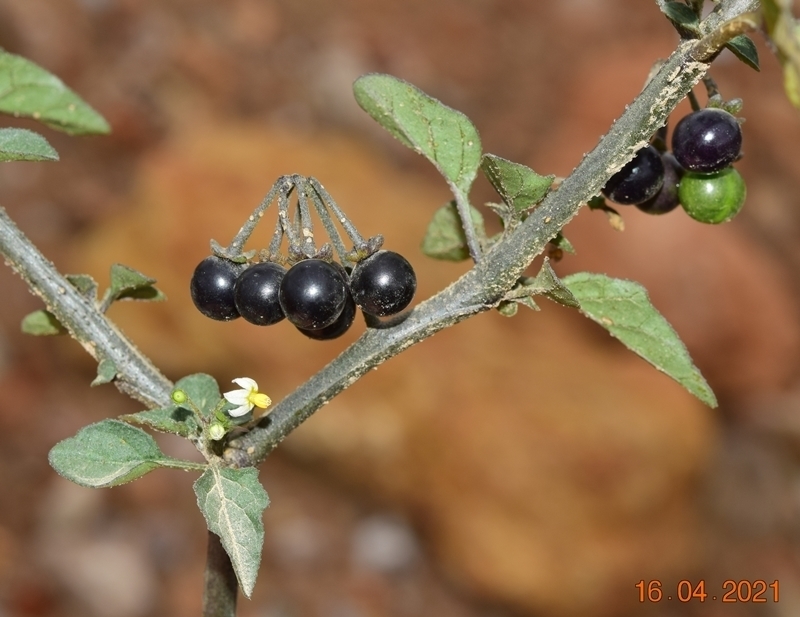
*Solanumnigrum* in flower and fruit, Almería, Spain (photo by Francisco Rodriguez, 16 April 2021). Source: https://www.inaturalist.org/observations/74174039 (iNaturalist 2021).

Calyx lobes more or less appressed (Särkinen et al. 2018); fruits green to black (Fig. 24); foliage dark green, blackish when dried (Kovalevskaya 1987).

#### Distribution

##### Native distribution

Southern Europe, Mediterranean, Southern Asia from Asia Minor to China. The species is most genetically diverse in Asia and may have an Asian origin (Särkinen et al. 2018). In Central Asia, the species is considered native in Turkmenistan, where it was recorded more commonly from riversides in the mountains (Pojarkova 1954b).

##### Secondary distribution

Archaeophyte in Boreal and Central Europe, Northern and Central Asia; neophyte in North America, South Africa, Malesia and Australia. One of the most common and widely distributed weeds in the world (Holm et al. 1979).

##### Distribution in Central Asia

Native in Turkmenistan, alien in Kazakhstan, Kyrgyzstan, Tajikistan and Uzbekistan.

Historically, *Solanumnigrum* was a common weed of irrigated fields (wheat, cotton, maize, maash) in Central Asia (Fedtschenko 1915).

Most of the major sources (e.g. Kovalevskaya 1987, Särkinen et al. 2018) do not make a distinction between the native and secondary distributions of *S.nigrum* because of the old age of its naturalisation in many areas. In this case, we considered type of habitat as the main distinguishing feature (Webb 1985) and assigned the alien status to the areas with overwhelmingly predominant ruderal occurrences.

##### Distribution in Kyrgyzstan

**Figure 25.**
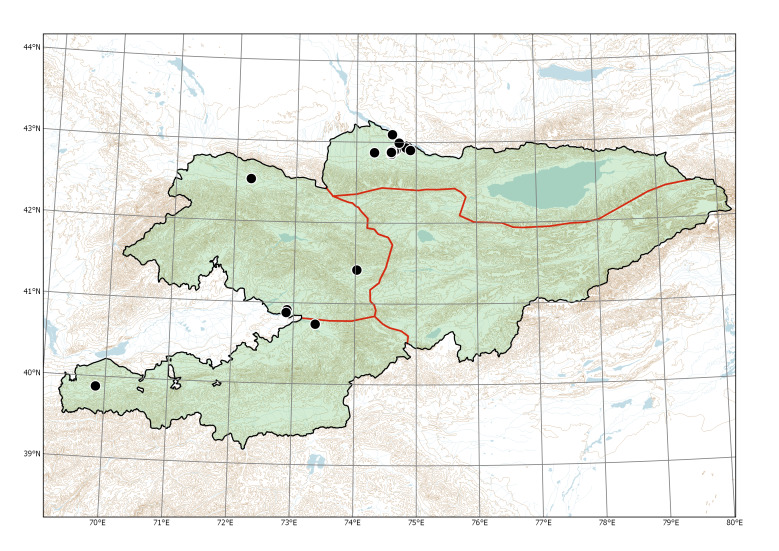
Recorded distribution of *Solanumnigrum* in Kyrgyzstan.

Western Tian-Shan, Northern Tian-Shan, Alay-Turkestan (Fig. 25).

The species was considered occurring in all parts of the country (Spota 1960, Deza 1983), although we have seen no collections from the Eastern Tian-Shan. It was collected at altitudes between 600 and 1350 m above sea level.

#### Ecology

Forest margins and riversides in the native distribution area; disturbed open places, cultivated lands, ruderal places, riversides in the secondary distribution area.

The species is capable of occurring successfully at altitudes above 2500 m, thus showing a high invasive potential also in the high mountains (Chandra Sekar et al. 2012).

#### Biology

Annual or short-lived perennial, with a taproot and numerous lateral roots.

#### Taxon discussion

*Solanumpseudoflavum* Pojark., with strongly reflexed calyx lobes and dark red fruits, was correctly synonymised with *S.olgae* Pojark. (= *S.villosum* Mill.) by Spota (1960) and Kovalevskaya (1987) but misplaced to the synonymy of *S.nigrum* by Särkinen et al. (2018). The latter synonymisation was adopted by PoWo (PoWo 2021).

#### Notes

Fruits and leaves of this species are not edible; reports of their use in Africa (e.g. Bvenura and Afolayan 2014, Essack et al. 2017) refer to *Solanumscabrum* Mill. and *S.villosum* Mill. (Särkinen et al. 2018, Sangija et al. 2021).

*Solanumnigrum* is likely an evolutionary derivative of *S.villosum* Mill. (Poczai and Hyvönen 2010); the distribution areas of both species are largely shared (Särkinen et al. 2018). Based on the presumably cultigenous origin of *S.villosum* (as explained under that species), we assume that the dispersal of *S.nigrum* was also partly human-mediated and this species was an unwanted component of the cultivation of *S.villosum*.

#### Introduction to Kyrgyzstan

##### Period of introduction

Archaeophyte.

This species was known from the whole of Central Asia from the beginning of its botanical exploration (Fedtschenko and Fedtschenko 1913). The time of its introduction is uncertain but is most likely Neolithic, as the species was recorded among the earliest and most common weeds in the early Neolithic of Germany (Rösch 1998).

##### Pathways of introduction

Transport - Contaminant: Seed contaminant.

The species is a noxious weed of gardens and fields (Deza 1983), also occurring in ruderal places (Spota 1960). It is capable of infesting a large variety of crops, including wheat and melons (e.g. Cherniakovskaya 1935, Ogg et al. 1981). We assume that *Solanumnigrum* arrived to the territory as a weed of historical crops (possibly of *S.villosum*).

Further dispersal occurs with water, contaminated seed and soil.

##### Invasion status

Naturalised, invasive. The species has been a noxious weed of all crops in Kyrgyzstan (Deza 1983) and remains common to date.

##### Evidence of impact

Agriculture - major impact (noxious weed of all crops, in fields and gardens). Native ecosystems - minor impact (occurring along streams and water bodies near populated places). Urban areas - major impact (ruderal occurrence).

##### Trend

Stable (observed).

### Solanum villosum

Mill. 1768

5FCA71F3-E92B-5F0F-B7D1-E89D55D7BBEB

urn:lsid:ipni.org:names:285344-2

Solanum villosum Mill., Gard. Dict., ed. 8: *Solanum* no. 2 (1768).
Solanum villosum *SolanumluteumSolanum*
Solanum villosum *Solanumolgae*
Solanum villosum *Solanumpseudoflavum*

#### Diagnosis

**Figure 26.**
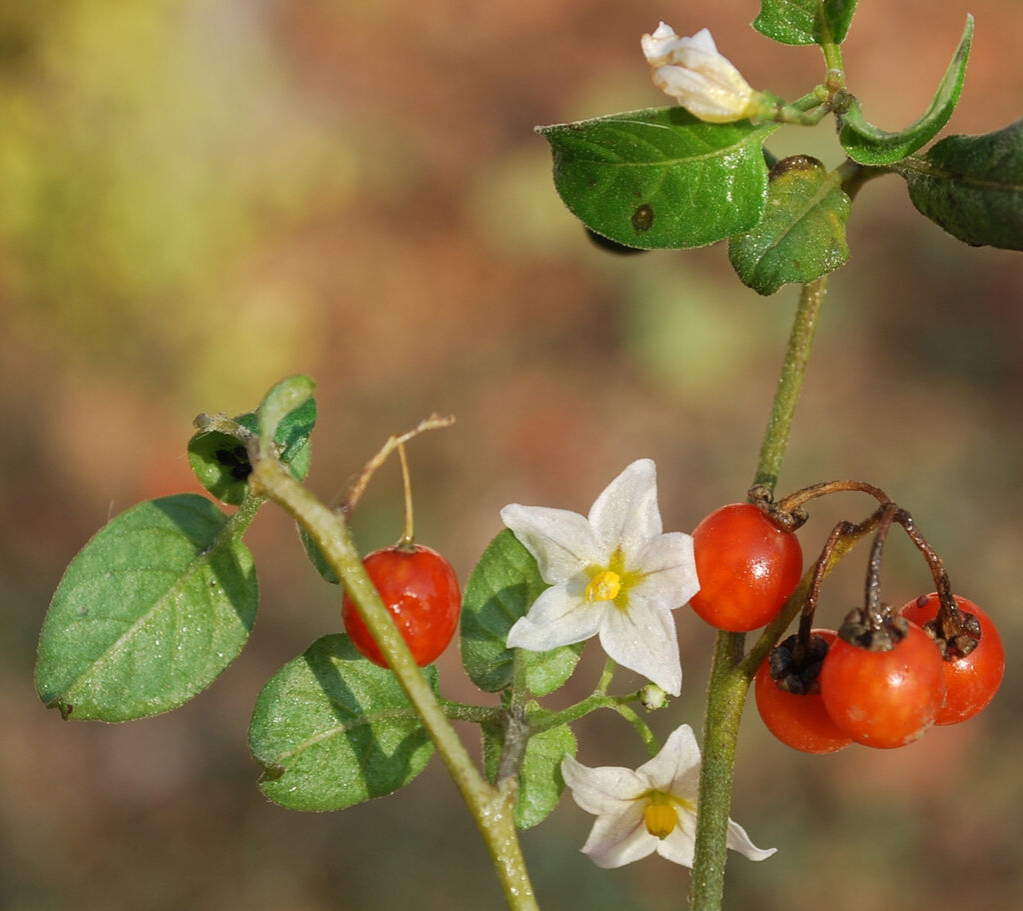
*Solanumvillosum* in flower and fruit in Tashkent, Uzbekistan (photo by T. Tillaev, 16 November 2011). Source: https://www.plantarium.ru/page/image/id/434617.html (Plantarium 2021).

Calyx lobes strongly reflected (Särkinen et al. 2018); fruits yellow, orange-red or dark red (Fig. 26); foliage green (Kovalevskaya 1987).

#### Distribution

##### Native distribution

Southern Europe, Mediterranean, Southern Asia from Asia Minor to China, Africa. In Central Asia, the species is considered native in Turkmenistan, where it was recorded abundantly from riversides in the mountains (as *Solanumnigrum* s.l.: Pojarkova 1954b).

##### Secondary distribution

Archaeophyte in Boreal and Central Europe, Northern and Central Asia and South Africa; neophyte in North America and Australia.

##### Distribution in Central Asia

Native in Turkmenistan, alien in Kazakhstan, Kyrgyzstan, Tajikistan, Uzbekistan.

Similarly to *Solanumnigrum*, we consider *S.villosum* as alien in Central Asia north of Turkmenistan because of its occurring exclusively on cultivated lands or in ruderal habitats (Spota 1960, Kovalevskaya 1987). Pojarkova (1955a) considered riversides as a native habitat of the species in the southern part of Central Asia.

##### Distribution in Kyrgyzstan

**Figure 27.**
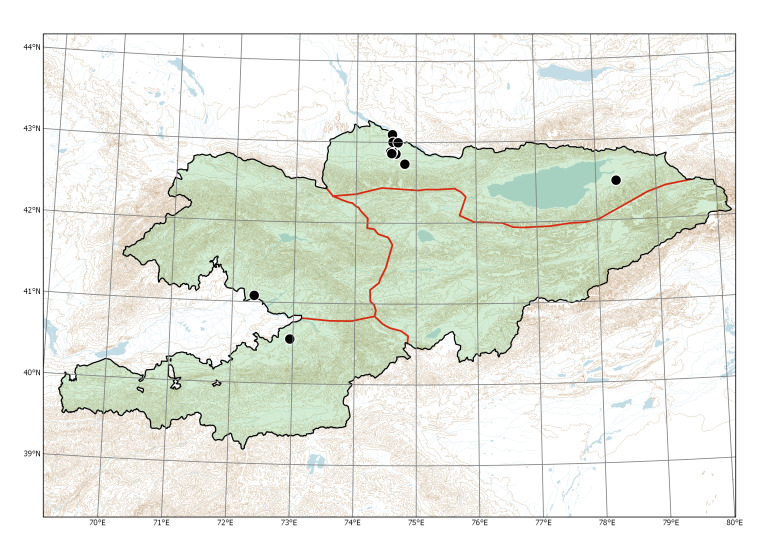
Distribution of *Solanumvillosum* in Kyrgyzstan.

Western Tian-Shan, Northern Tian-Shan, Alay-Turkestan (Fig. 27).

The species occurs in major agricultural territories: the Fergana Depression, the Chü Depression and the Ysyk-Köl Depression (Spota 1960, Lazkov and Sultanova 2014). It was collected at altitudes between 600 and 1800 m above sea level.

#### Ecology

Forest margins and riversides in the native distribution area; disturbed open places, cultivated lands, ruderal places, riversides in the secondary distribution area.

#### Biology

Annual or short-lived perennial, with a taproot and numerous lateral roots.

#### Taxon discussion

Pojarkova (1955a) and Pojarkova (1955b) subdivided *Solanumvillosum* in Central Asia into three narrowly defined species. Besides *S.luteum*, which Pojarkova recognised as a densely glandular variant, the plants without glandular pubescence were treated as *S.pseudoflavum* (leaves larger, subentire) and *S.olgae* (leaves smaller, repandly dentate). The latter two have already been synonymised by Spota (1960) and Kovalevskaya (1961).

The densely glandular variant occurs in scattered localities in Kazakhstan and Turkmenistan (Kovalevskaya 1987).

#### Notes

*Solanumvillosum* seems to be an evolutionary derivative of *S.americanum* Mill. (Poczai and Hyvönen 2010). Both species are edible and used by humans throughout their ranges (Särkinen et al. 2018). *Solanumamericanum* is native to the New World but distributed as native also in the tropical and subtropical Old World, i.e. Africa, India, Malesia and Australia; this distribution indicates that *S.americanum* may have been human-dispersed from the New World in prehistorical times, similarly to (or together with) *Daturametel* L. (Geeta and Gharaibeh 2007). Since *S.villosum* may have originated directly from *S.americanum* by autoallopolyploidisation (Poczai and Hyvönen 2010), its ancient cultigenous origin and human-mediated dispersal is quite likely.

#### Introduction to Kyrgyzstan

##### Period of introduction

Archaeophyte.

This species was known from the whole of Central Asia from the beginning of its botanical exploration (Pojarkova 1955b). The time of its introduction is uncertain.

##### Pathways of introduction

Escape from confinement: Agriculture.

The species is especially abundant in Southern Europe, Central and Southern Asia and Africa, where it has been traditionally used as a fruit or leaf vegetable (Cherniakovskaya 1935, Pojarkova 1955a, Baranov 1967, Defelice 2003, Njau Mwai et al. 2007, Särkinen et al. 2018). It was known in Chinese cultivation in the Russian Far East during the 19th century (Schischkin 1936). For this reason, we assume that the species was introduced intentionally as a cultivated plant, rather than unintentionally as a weed. Its current weedy status seems to be secondary.

Further dispersal occurs with water, contaminated seed and soil, in the same way as *Solanumnigrum*.

##### Invasion status

Naturalised. The species was noted as a weed in gardens and on fields, locally common but seemingly not truly noxious (Spota 1960).

##### Evidence of impact

Agriculture - major impact (locally common weed of crops, in fields and gardens). Native ecosystems - minor impact (occurring along streams and water bodies near populated places). Urban areas - moderate impact (ruderal occurrence).

##### Trend

Stable (inferred).

## Discussion

The variety of plants recorded for the present Contribution, ranging from the oldest archaeophytes to the most recent neophytes, reflect the long and complicated history of the human civilisation in Central Asia.

Some archaeophytes in Central Asia are notably old, dating back to the Neolithic period. This is true for old cultivated plants (*Alkekengiofficinarum*, *Solanumvillosum*) and their weeds (*Solanumnigrum*). The period of Islamic states of Transoxiana (Mavarannahr), probably from the beginning of the 11th century, was noted for introductions of early medicinal (*Daturastramonium* and *Hyoscyamusniger*) and ornamental (*Hemerocallisfulva*) plants. The period of the Russian conquest of Central Asia (late 19th century) brought other cultivated plants that quickly became ruderal, i.e. *Nicandraphysalodes* which was used in home bakery as a substitute for yeast.

The recent period is characterised by the intensive import of foreign grain and seed for cultivation and consumption. Due to this recent import, *Physalisphiladelphica* was introduced to Central Asia (first recorded in 2008) and may become a common weed in the future.

The latest increase (expansion and diversification) of ornamental cultivation brings the risk of further introductions of unwanted plants (cf. Dehnen‐Schmutz et al. 2007, van Kleunen et al. 2018a); this process is reflected in our first record of *Daturainnoxia* and the renewed record of *Nicandraphysalodes* outside their places of cultivation. As the ornamental cultivation is still actively developing in Kyrgyzstan, we expect more records of such plants in the future.

Among the old archaeophytes, a prominent decline was observed in the occurrence of *Alkekengiofficinarum* due to the decrease of its cultivation. A similar level of decline was also observed in Tajikistan (Nowak et al. 2014). A strong decline was also suggested for *Solanumvillosum* in Tajikistan (Nowak et al. 2014); although this species is rather rare nowadays, it can still be found in and around villages in Kyrgyzstan, and its ruderal occurrence in Tashkent indicates the presence of viable populations in Uzbekistan (observations on Plantarium 2021). No decline or rarity was observed for *Solanumnigrum*.

The oldest introductions may disappear without any signs in the recent flora. According to the latest archaeological research, developed agriculture existed in the Ysyk-Köl Depression approximately 3000 years ago, in the transition period from the Late Bronze Age to the Early Iron Age (Motuzaite Matuzeviciute et al. 2021). Most notably, *Buniasorientalis* L. and *Glebionis* sp. were found in the site as early vegetables, thus shifting the history of use of the first species deeper into prehistoric times. *Buniasorientalis* was found used as a vegetable also in prehistoric Southern Siberia, but its presence in the ancient cultivation did not affect the recent flora until the species' arrival as a grain contaminant at the turn of the 19th and 20th centuries (Sennikov and Lazkov 2021). *Glebioniscoronaria* and *G.segetum* are still used as leaf vegetables in China (Li 1970), most probably introduced through India due to their southern areas of cultivation (Shih et al. 2011).

Among the plants introduced during the period of Islamic states in Central Asia, the former medicinal plants (*Daturastramonium* and *Hyoscyamusniger*) became weeds and ruderals; their position in the flora is stable. The old ornamental plant, *Hemerocallisfulva*, is seemingly extinct in the wild, as well as the introduction of the period of the Russian colonisation, *Nicandraphysalodes*, is no longer present in the places of its former cultivation. However, the old introduced plants, once having gone out of fashion and therefore extinct, may be reintroduced into cultivation and found in the wild again; both of the aforementioned plants, *H.fulva* and *N.physalodes*, have experienced the reintroduction from Europe, and the latter species has been lately seen in the wild again.

There are special cases when certain plant species, native to the Americas, were found introduced to the Old World, probably originally to India or other territories nearby, in the pre-Columbian era. Among such introductions, the history of *Daturastramonium* is best studied (Geeta and Gharaibeh 2007). *Solanumvillosum* also belongs to the group of American origin (Poczai and Hyvönen 2010), whose distribution in the Old World may be also considered secondary (human-mediated) but sufficiently old to form extensive areas.

Regarding the difficulties encountered during this compilation, most notable was the lack of dedicated scientific studies and the paucity of herbarium collections, which reflect the current situation in plant invasions completely inadequately. Thanks to the recent development of online observational facilities and citizen-science tools (e.g. Plantarium 2021, iNaturalist 2021), this deficiency is perfectly compensated by observations documented with photographs. As a rule, herbarium specimens provide a valuable source of information on early plant invasions, whereas the recent waves of plant introductions and the current distributions of plant species may be reflected largely or even exclusively in observations. This situation corresponds to the usage of material in regular plant mapping projects, in which herbarium collections have already lost part of their traditional role (cf. Khapugin et al. 2021).

Another issue is the incompleteness of older publications and the poor availability of their background data. Quite exemplarily, the historical record of *Nicandraphysalodes* dated back to 1928 (the undated mention in Cherniakovskaya (1935)), but it was published as actual in Nikitina (1960) and maintained without comments in more recent publications (Kovalevskaya 1987, Lazkov and Sultanova 2011, Lazkov and Sultanova 2014), thus making an impression that the record may reflect the current situation. However, the uncovered background data clearly demonstrate that this record is linked with historical drivers which have ceased to exist already long ago, and the current presence of the species in Kyrgyzstan (only recently confirmed) is different and must be linked with another, contemporary factor.

## Supplementary Material

XML Treatment for Alkekengi officinarum

XML Treatment for Bidens frondosa

XML Treatment for Datura innoxia

XML Treatment for Datura stramonium

XML Treatment for Hemerocallis fulva

XML Treatment for Hyoscyamus niger

XML Treatment for Nicandra physalodes

XML Treatment for Physalis angulata

XML Treatment for Physalis philadelphica

XML Treatment for Solanum nigrum

XML Treatment for Solanum villosum
